# Mitochondrial Transplantation Modulates Inflammation and Apoptosis, Alleviating Tendinopathy Both In Vivo and In Vitro

**DOI:** 10.3390/antiox10050696

**Published:** 2021-04-28

**Authors:** Ji Min Lee, Jung Wook Hwang, Mi Jin Kim, Sang Youn Jung, Kyung-Soo Kim, Eun Hee Ahn, Kyunghoon Min, Yong-Soo Choi

**Affiliations:** 1 Department of Biotechnology, CHA University, Seongnam 13488, Korea; mjl10002@naver.com (J.M.L.); hellenic1019@gmail.com (J.W.H.); treasure7744@naver.com (M.J.K.); 2 Division of Rheumatology, Department of Internal Medicine, CHA Bundang Medical Center, CHA University School of Medicine, Seongnam 13496, Korea; jungsy7597@chamc.co.kr; 3 Division of Endocrinology and Metabolism, Department of Internal Medicine, CHA Bundang Medical Center, CHA University School of Medicine, Seongnam 13496, Korea; kks982@chamc.co.kr; 4 Department of Obstetrics & Gynecology, CHA Bundang Medical Center, CHA University School of Medicine, Seongnam 13496, Korea; bestob@chamc.co.kr; 5 Department of Rehabilitation Medicine, CHA Bundang Medical Center, CHA University School of Medicine, Seongnam 13496, Korea

**Keywords:** inflammation, apoptosis, mitochondrial dynamics, mitochondrial transplantation, tenocyte, tendinopathy

## Abstract

Tendinopathy is a common musculoskeletal condition causing pain and dysfunction. Conventional treatment and surgical procedures for tendinopathy are insufficient; accordingly, recent research has focused on tendon-healing regenerative approaches. Tendon injuries usually occur in the hypoxic critical zone, characterized by increased oxidative stress and mitochondrial dysfunction; thus, exogenous intact mitochondria may be therapeutic. We aimed to assess whether mitochondrial transplantation could induce anti-inflammatory activity and modulate the metabolic state of a tendinopathy model. Exogenous mitochondria were successfully delivered into damaged tenocytes by centrifugation. Levels of Tenomodulin and Collagen I in damaged tenocytes were restored with reductions in nuclear factor-κB and matrix metalloproteinase 1. The dysregulation of oxidative stress and mitochondrial membrane potential was attenuated by mitochondrial transplantation. Activated mitochondrial fission markers, such as fission 1 and dynamin-related protein 1, were dose-dependently downregulated. Apoptosis signaling pathway proteins were restored to the pre-damage levels. Similar changes were observed in a collagenase injection-induced rat model of tendinopathy. Exogenous mitochondria incorporated into the Achilles tendon reduced inflammatory and fission marker levels. Notably, collagen production was restored. Our results demonstrate the therapeutic effects of direct mitochondrial transplantation in tendinopathy. These effects may be explained by alterations in anti-inflammatory and apoptotic processes via changes in mitochondrial dynamics.

## 1. Introduction

Tendinopathies are common musculoskeletal problems associated with aging and sports [1]. Rotator cuff, lateral elbow, Achilles, and patellar tendons are commonly affected areas [2]. Patients with tendinopathies suffer from activity-related pain and dysfunction [3]. There are conflicting results regarding the efficacies of conventional treatments for tendinopathies, such as physical modalities, corticosteroid injection, and analgesic medications [4,5]. The surgical repair of ruptured tendons has not been satisfying, especially in the elderly population [6]. The pathogenesis of tendinopathy involves interactions between processes related to degeneration and inflammation [7,8]. Primarily, inflammation is reportedly involved in the full spectrum of tendinopathies [9]. Since tendon injuries mainly occur in the “critical zone”, an area with insufficient blood supply, their healing is not complete [10,11]. Hypoxia-inducible factor (HIF)-α is increased in the rotator cuff hypoxic zone [12]. A hypoxic environment induces mitochondrial dysfunction, promotes inflammation, and alters the metabolic profile via increased oxidative stress [13]. Hypoxia is a critical regulator of tendinopathy, causing inflammation and apoptosis in torn tendons in humans; thus, hypoxic cell injury is a novel therapeutic target [14]. Antioxidants targeting mitochondria show protective effects on human tendon cell damage [15]. Furthermore, a hypoxic state dysregulates mitochondrial homeostasis, resulting in high levels of reactive oxygen species (ROS), nuclear factor (NF)-κB dimerization, and mitophagy [16,17]. Activation of HIF-α under hypoxic conditions leads to the inhibition of mitochondrial biogenesis via PGC1-α (peroxisome proliferator-activated receptor-gamma coactivator1-α) suppression [18].

Mitochondrial dysfunction has been observed in a broad spectrum of diseases, and studies of therapies using or targeting mitochondria have increased exponentially [19,20]. Clinically, mitochondrial transplantation has been suggested as a potential treatment for incurable or mitochondrial diseases such as cardiomyopathy or Pearson bone marrow syndrome (NCT03384420, clinicaltrials.gov) [21,22]. However, therapies directly focusing on mitochondria have not been reported for tendinopathy, to the best of our knowledge. Mitochondria-targeted therapies can be categorized into mitochondrial modulation and transplantation strategies [23]. Although mitochondria are a source of damage-associated molecular patterns, the transplantation of mitochondria results in the recovery of damaged tissues and cellular metabolism [16,23,24]. In this study, the effects of the direct mitochondrial transfer on tendinopathy were evaluated. Moreover, we aimed to investigate its underlying mechanisms.

## 2. Materials and Methods

### 2.1. Cell Culture

This in vitro study was approved by the CHA University Institutional Review Board (IRB number: NON2018-003). Umbilical cord-mesenchymal stem cells (UC-MSCs) were obtained from the Wharton’s jelly as in our previous study [25]. Briefly, Warton’s jelly of umbilical cords was sliced into 5 mm explants. The slices were cultured in minimum essential medium Eagle-alpha modification (α-MEM; HyClone Laboratories Inc., Logan, UT, USA) supplemented with 10% fetal bovine serum (FBS; HyClone Laboratories Inc., Logan, UT, USA). UC-MSCs from UC fragments were expanded. For this experiment, isolated UC-MSCs were cultured in a T-175 flask with α-MEM (HyClone) supplemented with 10% FBS (HyClone), and 100 IU/mL penicillin, 100 mg/mL streptomycin (P/S; HyClone) at 37 °C in a humidified atmosphere containing 5% CO_2_. Human tenocytes (#TEN-F; ZenBio Inc.; Durham, NC, USA) and L6 myoblasts (CRL-1458; Animal Type Culture Collection; Manassas, VA, USA) were purchased. Human tenocytes and rat cells (L6 myoblasts) were cultured in Dulbecco’s Modified Eagle Medium (HyClone) supplemented with 10% FBS (HyClone) and 1% P/S (HyClone) at 37 °C in a humidified atmosphere containing 5% CO_2_. Tenocytes were cultured on pre-coated flasks/plates with collagen type 1 (C 8919; Sigma, St. Louis, MO, USA) for 24 h and rinsed with 1× Dulbecco’s phosphate-buffered saline (DPBS; Welgene Inc., Gyeongsan, Gyeongsangbuk-do, Korea) prior to seeding cells. UC-MSCs (passages 5–7), tenocytes (passages 3 or 4), and L6 myoblasts (passages 5 or 6) were used. Those cells were subcultured when they reached approximately 80–90% confluence.

### 2.2. Establishment of a Damaged Tenocyte Model

Recombinant human tumor necrosis factor-α (TNF-α) was purchased from PeproTech (No. 300-01A; Rocky Hill, NJ, USA). TNF-α was dissolved with pure ethanol to prepare a 20 µg/mL stock stored at 4 °C. The test concentrations used were 10, 50, 100, and 500 ng/mL in a growth medium. Tenocytes were finally treated with TNF-α (10 ng/mL) for 24 h to establish an in vitro tendinopathy model (Supplementary Figure S1).

### 2.3. Isolation of Mitochondria

UC-MSCs and the L6 rat myoblast cell line were used for in vitro and in vivo experiments, respectively. Mitochondria were separated by mechanical homogenization and differential centrifugation. After adding 400 μL of SHE buffer [0.25 M sucrose, 20 mM HEPES (pH 7.4), 2 mM EGTA, 0.1% defatted bovine serum albumin] to a 2 × 10^7^-cell suspension, cells were mechanically disrupted using a 1 mL syringe (100 strokes) (Korea vaccine, Seoul, Korea). To remove unbroken cells and nuclei, centrifugation was performed at 1100× *g* for 3 min at 4 °C. The supernatant was collected and centrifuged at 12,000× *g* for 15 min. To wash the crude separated mitochondria, the pellet was suspended in 1.5 mL of SHE buffer and then centrifuged at 20,000× *g* for 10 min. After removing the supernatant, the final mitochondrial pellet was suspended in 1.5 mL of DPBS (Welgene) and centrifuged at 20,000× *g* for 5 min. After removing the supernatant, the mitochondrial pellet was suspended in 200 μL of DPBS (Welgene); 10 μL of the mitochondrial solution was obtained, and the concentration was determined using a bicinchoninic acid assay (Pierce, Rockford, IL, USA) (Supplementary Figure S2A).

### 2.4. Transfer of Isolated Mitochondria on Tenocytes In Vitro

Mitochondria from UC-MSCs were transferred into tenocytes pretreated with TNF-α by centrifugation [26]. In brief, tenocytes were added to 100 μL of PBS on ice. The mitochondria doses (1, 5, 25 μg) refer to the weight of donor cell mitochondria per 1 × 10^5^ tenocytes. Isolated mitochondria were mixed slowly with tenocytes stored in PBS. The mixture was centrifuged at 1500× *g* for 5 min and washed with PBS two times. Then, cells were seeded into a plate according to the experimental design. This process allowed the transfer of mitochondria to tenocytes. All subsequent analyses were performed 24 h after mitochondrial transfer. 

### 2.5. Verification of the Incorporation of Exogenous Mitochondria into Tenocytes

MitoTracker Green (Thermo Fisher, Waltham, MA, USA) and MitoTracker CMXRos red (CMXRos red) (Thermo Fisher) were used to confirm the mitochondrial transfer to tenocytes by fluorescence microscopy. First, tenocytes were seeded (1 × 10^5^ cells/well) into a 6-well plate, and UC-MSCs were seeded (1 × 10^5^ cells/flask) into a T-175 culture flask. Tenocytes were treated with TNF-α at 10 ng/mL for 24 h. After 24 h, for visualization of mitochondria incorporation, damaged tenocytes and donor cells were stained with MitoTracker Green or CMXRos red, respectively, according to the manufacturer’s protocols. The cells were washed with Hank’s balanced salt solution (HyClone) and cultured with 300 nM MitoTracker Green and CMXRos red at 37 °C and 5% CO_2_ for 30 min. Then, mitochondria were isolated from donor cells, and extracted mitochondria were transferred into tenocytes by centrifugation. After adding 4′6-diamidino-2-phenylindole (DAPI; Vector, Burlingame, VT, USA) to tenocytes, slides were prepared (Supplementary Figure S2B). A 100× image of the cells was obtained using a fluorescence microscope (Leica Microsystems, Mannheim, Germany).

### 2.6. Establishment of an In Vivo Tendinopathy Model

This animal study proceeded after approval from the Institutional Animal Care and Use Committee at CHA University (IACUC190102). Twenty-one male Sprague–Dawley rats (age, 5 weeks; weight, 100–150 g) were randomly distributed into the following four groups: negative control (*n* = 3), positive control (*n* = 6), low mitochondrial concentration (*n* = 6), and high mitochondrial concentration (*n* = 6) groups. The Achilles tendons of both hind limbs were injected with 0.6 mg/40 μL collagenase (Sigma-Aldrich, Dorset, UK). After 2 weeks, isolated mitochondria from L6 cells were injected into the tendon by local injection. Bodyweight was the weight on an electronic scale, and limb thickness was measured using a caliper every week. Animals in each group were sacrificed using carbon dioxide (CO_2_) after 2 weeks to evaluate the efficacy of mitochondrial transplantation.

### 2.7. Injection of Isolated Mitochondria In Vivo

In animal models, mitochondria were isolated from L6 cells following the methods used for the previous in vitro analysis. Briefly, L6 cells were mechanically homogenized, and mitochondria were purified by differential centrifugation (Supplementary Figure S2A). Isolated mitochondria were stored in DPBS (Welgene) and injected locally into the damaged tendon using a 29-gauge syringe at 10 µg (low) and 50 µg (high) in a 20 µL volume. For the experiment to confirm the successful mitochondrial delivery, L6 cells were stained with CMXRos red reagent before mitochondrial isolation.

### 2.8. Confirmation of Mitochondrial Transplantation In Vivo

Tendon tissues were examined to visualize delivered mitochondria after local mitochondrial injection. Stained mitochondria from L6 cells with CMXRos red were injected locally into the Achilles tendon. Tissue samples were used for cryosectioning. Right after harvesting, the tendon tissue was fixed in 4% paraformaldehyde (BP031, Bio-solution, Suwon-si, Gyeonggi-do, Korea) with 4 °C overnight. After rinsing the tissue with 1× PBS, the fixed material was shaken with 10% and 20% sucrose for 3 h at 4 °C and overnight with 30% sucrose at 4 °C, then we waited until the tissue subsides in a 50 mL conical tube. After making a square mold with foil, the OCT compound (Sakura, 4583, Torrance, CA, USA) was added appropriately, and then the tissue was embedded and frozen rapidly on nitrogen without bubbles. After that, the compound should be stored at −70 °C. The tissues were cut to 5 μm thick by the cryostat (CM3050S, Leica Microsystems) at −20 °C. The temperature of the cutting chamber was occasionally adjusted ±2 °C. After attaching sections to a slide, 300 nM MitoTracker Green dye was added to the sections for 30 min at 37 °C and 5% CO_2_; this was followed by DAPI staining. Fluorescence images at 400× magnification were obtained using a confocal laser scanning microscope ZEISS LSM 880 (Carl Zeiss AG, Oberkochen, Germany).

### 2.9. ROS Measurement

Intracellular ROS was measured using the 2′,7′-dichlorofluorescein diacetate (DCF-DA) probe (Molecular Probes, Eugene, OR, USA). Cells were seeded in a 48-well plate at a concentration of 1 × 10^4^ cells per well and then cultured in a growth medium for 24 h at 37 °C and 5% CO_2_. When cells reached about 80–90% confluence, they were treated with TNF-α, and mitochondria were delivered. After 24 h, cells were washed once with PBS and incubated for 30 min with 10 μM DCF-DA. Fluorescence intensity was measured at an excitation wavelength of 530 nm and an emission wavelength of 590 nm using a Synergy Mix Multi-Mode Reader (BioTek Inc., Winooski, VT, USA).

### 2.10. Mitochondrial ROS (mROS) Measurement

mROS was measured using MitoSOX Red, a mitochondrial superoxide indicator (Invitrogen, Carlsbad, CA, USA). Cells were seeded in a 48-well plate at a concentration of 1 × 10^4^ cells/well and then cultured in a growth medium for 24 h at 37 °C and 5% CO_2_. When cells reached about 80–90% confluence, they were treated with TNF-α and mitochondria were delivered. After 24 h, cells were washed with once with PBS and incubated for 30 min with 1 μM MitoSOX Red at 37 °C and 5% CO_2_. Fluorescence intensity was measured at an excitation wavelength of 510 nm and an emission wavelength of 528 nm using a Synergy Mix Multi-Mode Reader (BioTek Inc.). Moreover, images of mROS level at 200× magnification were obtained using a fluorescence microscope (Leica Microsystems).

### 2.11. Adenosine Triphosphate (ATP) Measurement

ATP was measured using the CellTiter-Glo Luminescence Kit (Promega, Madison, WI, USA). Growth medium (100 μL) and CellTiter-Glo Luminescence Test Solution (100 μL) were added to cells treated with TNF-α and mitochondria, and the treated cells were left first for 10 min and then for 2 min with shaking at room temperature (22 °C–23 °C). Emission signals were measured using a Luminescence microplate reader (Epoch Spectrometer, BioTek Inc.).

### 2.12. Mitochondrial Membrane Potential

A JC-1 assay was performed. When JC-1 dye accumulates in healthy mitochondria, emission shifts from green (528 nm) to red (590 nm). The JC-1 dye was stored at 7.7 mM after dilution in dimethyl sulfoxide. The growth medium was supplemented with 2 µM JC-1 immediately before the experiment. Cells were incubated for 20 min at 37 °C and then washed two times with DPBS (Welgene). Then, cells in DPBS (100 µL) (Welgene) were analyzed using a microplate reader (Epoch Spectrometer, BioTek Inc.).

### 2.13. Real-Time Polymerase Chain Reaction (PCR) Analysis

Gene expression levels of Tenomodulin (*Tnmd*), Collagen 1 (*CoL1*)*,* and matrix metalloproteinase 1 (*MMP1*) were analyzed by qPCR (Supplementary Table S1). Total RNA was isolated using an Easy-spin Total RNA Extraction Kit (iNtRON Biotechnology Inc., Seoul, Korea) according to the provided protocol. cDNA synthesis was performed with 1 μg of total RNA using the Maxime RT PreMix Kit (iNtRON Biotechnology Inc.; No. 25081). Briefly, 1 μg of RNA was placed in each tube and auto-distilled water was added to obtain a final volume of 20 μL. The PCR steps were repeated for 25 cycles under the following conditions: pre-denaturation at 95 °C for 5 min, denaturation at 95 °C for 30 s, annealing at 58 °C for 45 s, elongation at 72 °C for 1 min, and final elongation at 72 °C for 5 min. Products were loaded on a 1.5% agarose gel and analyzed under ultraviolet light. Then, real-time PCR was performed using SYBR Green to quantify gene transcripts, and amplification products were analyzed using the StepOnePlus System (Applied Biosystems, Inc., Waltham, MA, USA). The housekeeping gene *GAPDH* was used as a control to normalize gene expression levels.

### 2.14. Western Blot Analysis

After treatment with TNF-α and the transplantation of mitochondria, cells were cultured at 37 °C under 5% CO_2_ for 24 h. Protein was extracted using RIPA buffer (100 μL per well) with protease inhibitor. After centrifugation at 13,572× *g* for 15 min at 4 °C, the supernatant was collected. The protein contents were determined by the BCA assay using an ELISA microplate reader at 562 nm. The proteins contents were normalized so that all samples had the same concentration and volume for further analysis. Each sample was heated with SDS-polyacrylamide gel electrophoresis (PAGE) sample buffer (62.5 mM Tris-HCl pH 6.8, 2% SDS, 10% glycerol, and 5% 2-mercaptoethanol) at 100 °C for 3 min. Proteins were separated by 12% SDS–PAGE and transferred to a PVDF membrane at 0.35 mA for 3 h. PVDF was blocked in pure methanol before use. The membrane was blocked in 1× TBS containing 3% bovine serum albumin (BSA; Bio Basic Inc., Markham, ON, Canada) and 0.1% Tween-20 for 1 h at room temperature. Membranes were incubated overnight with the following primary antibodies diluted at a 1:1000 ratio in 3% BSA: anti-BID (Santa Cruz Biotechnology, Santa Cruz, CA, USA; FL-195), anti-Bax (Santa Cruz; sc-7480), anti-Bcl2 (Santa Cruz; sc-7480), anti-Cytochrome C (Santa Cruz; sc-13156), anti-Tenomodulin (TNMD) (Abcam, Cambridge, UK; ab203676), anti-Tenascin C (TNC) (Abcam; ab108930), anti-Collagen 1 (COL1) (Abcam; ab6308), anti-matrix metalloproteinase 1 (MMP1) (Santa Cruz; sc-58377), p-NF-kB p65 (Ser536), NF-kB p65 (Cell Signaling, Danvers, MA, USA; 9663S), anti-Fis1 (Santa Cruz; sc-376446), anti-Drp1 (Abcam; ab56788), anti-Mitofusin 2 (MFN2) (Abcam; ab56889), total OXPHOS rodent (Abcam; ab110413), and anti-β-actin (Santa Cruz; sc-4778) antibodies. After the membranes were washed three times for 10 min with TBS-T, they were reacted with a secondary antibody conjugated with horseradish peroxidase (HRP), goat anti-mouse antibody (Santa Cruz Biotechnology Inc.; sc-2005) or mouse anti-rabbit antibody (Santa Cruz; sc-2357), at a dilution of 1:5000 in 3% BSA in TBS-T for 1 h at room temperature. After washing membranes three times with TBS-T for 10 min, signals were visualized by enhanced chemiluminescence (ECL component from Pierce Clarity and Western ECL Substrate from Bio-Rad, Hercules, CA, USA), and detected using a LAS-4000 (Fuji Photo Film Co., Tokyo, Japan). Uncropped raw images of all blots were included in Supplementary Figure S3.

### 2.15. Cytokine Assay

To analyze IL-6 and IL-1β activities, the culture medium was collected 24 h after stimulation of TNF-α and transfer of mitochondria. Cells were centrifuged at 212× *g* for 5 min. The supernatant was harvested and analyzed. IL-6 and IL-1β were analyzed according to the manufacturer’s instructions provided with the ELISA kit (BioSource International, Camarillo, CA, USA). Next, IL-6 and IL-1β standard proteins, analysis buffer, and biotin-binding detection antibody were added to the cells, and the cells were cultured for 24 h. After incubation, each plate was washed with PBS and incubated with streptavidin-HRP for 1 h. After the color reaction with a TMB solution, absorbance was measured at 450 nm using an Epoch spectrometer/microplate reader (BioTek Inc.).

### 2.16. Microarray Analysis

Tenocytes were seeded at 1 × 10^5^ cells per well in a 6-well plate and treated with TNF-α (10 ng/mL) for 24 h. Then, the isolated mitochondria were transferred, and RNA was extracted after 24 h using the Easy-spin Total RNA Extraction Kit (iNtRON Bio) and a NanoDrop spectrophotometer (Thermo Fisher) (260:280 > 1.9 and 260:230 > 1.9). The mitochondrial concentration (yield) was determined to be 1 μg, and the purity was measured. Microarray analysis was performed using Affymetrix HuGene 2.0ST Chips (Affymetrix, Santa Clara, CA, USA). A heatmap clustering analysis was performed using Morpheus.

### 2.17. Hydroxyproline Assay

A hydroxyproline assay was performed to confirm the total tendon collagen content. The sacrificed tissue was rapidly frozen in liquid nitrogen and stored at −80 °C. Tissue disruption and experimental methods were performed following the manufacturer’s instructions for a hydroxyproline colorimetric assay (Abcam; ab222941). After breaking the tissue, proteins were treated with 10 N NaOH and HCl and then centrifuged at 10,000× *g* for 5 min. After collecting the supernatant, an oxidation mixture was added and reacted at room temperature for 20 min. The developer and DMAB concentrate were added at 50 μL per vial and reacted at 65 °C for 45 min. Measurements were obtained at 560 nm using a Synergy Mix Multi-Mode Reader (BioTek).

### 2.18. Mdivi-1 Treatment

The inhibitor Mdivi-1 was processed to confirm the impact of mitochondria fission. Mdivi-1 (M0199, Sigma-Aldrich) was treated on tenocytes in DMEM (HyClone) supplemented with 10% FBS (HyClone) for 24 h with TNF-α treatment. After 24 h of mitochondrial transplantation, western blot analysis was performed.

### 2.19. Statistical Analysis

All statistical analyses were performed using SigmaPlot 11.0 (Systat, San Jose, CA, USA). The significance of differences between groups was evaluated by one-way analysis of variance, with post hoc tests for multiple comparisons. Quantitative results are shown as mean ± SEM values, and statistical significance was defined at *p* < 0.05. 

### 2.20. Data Availability

The data that support the findings of this study are available from the corresponding authors upon reasonable request. 

## 3. Results

### 3.1. Successful Mitochondrial Isolation and Transplantation into Tenocytes Confirmed by Expression of Mitochondrial Markers In Vitro

**Figure 1:**
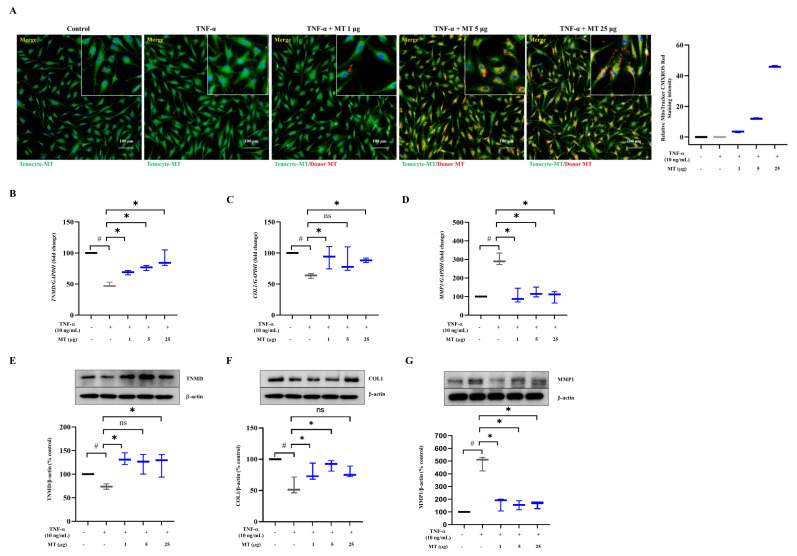
Exogenous mitochondria successfully transplanted into damaged tenocytes enhance tenocyte-related marker levels and decrease MMP1 level. (**A**) Representative fluorescence images of tenocytes showing endogenous mitochondria (MitoTracker Green) and increased doses (1, 5, 25 μg) of exogenous mitochondria (MitoTracker CMXR_OS_ red) with DAPI nuclear staining (blue). Red fluorescent signal intensity increased dose dependently. (**B**–**D**) Gene expression of (**B**) *TNMD*, (**C**) *COL1*, and (**D**) *MMP1* (*GAPDH* as loading control). (**E**–**G**) Representative western blots and densitometric quantification of (**E**) TNMD, (**F**) COL1, (**G**) MMP1 (β-actin as loading control). Data represent mean ± standard deviation (SD) (*n* = 3). # *p* < 0.05 between TNF-α (+) group (black) and TNF-α (−) group as control (white), * *p* < 0.05 between MT (+) group damaged by TNF-α (gray) and MT (−) group damaged by TNF-α (black) TNF-α, tumor necrosis factor-α; MT, mitochondria; TNMD, tenomodulin; COL, collagen; MMP1, matrix metalloproteinase-1.

Isolated mitochondria from UC-MSCs were then evaluated using mitochondrial markers (apoptosis-inducing factor, translocase of the outer membrane, complex IV, and cytochrome C; isolated mitochondria versus remaining cytosol fractions) (Supplementary Figure S4A). Isolated intact mitochondria could maintain their membrane potential through ATP production, indicating that their function was preserved, unlike that of physically ruptured mitochondria (Supplementary Figure S4B–E). Exogenous mitochondria were delivered into damaged tenocytes by centrifugation, as described in our previous study [26]. The mitochondria of tenocytes and UC-MSCs were stained with MitoTracker Green and CMXRos red fluorescent dyes for endogenous (green) and exogenous (red) mitochondrial detection, respectively. Yellow color indicated merging of two colors (green and red). The staining results showed that mitochondrial transplantation was successful. The red signal tended to increase as exogenous mitochondria increased (Figure 1A and Supplementary Figure S5).

### 3.2. Mitochondrial Transplantation Enhanced TNMD and COL1 Expression and Decreased MMP1 Expression in TNFα-Treated Tenocytes In Vitro

We investigated tenocyte-related markers (TNMD and COL1) and MMP1 after the mitochondrial transfer [27,28]. The expression of *TNMD,* the most specific tenocyte marker reported to date [27], decreased by about 51% upon exposure to 10 ng/mL TNF-α for 24 h (*p* < 0.05, versus without TNF-α exposure) and recovered at 24 h after mitochondrial transfer in a dose-dependent manner (1, 5, and 25 µg; *p* < 0.05, versus TNF-α exposure without mitochondrial transfer) (Figure 1B). *COL1* expression was also suppressed by about 37% in damaged tenocytes (10 ng/mL TNF-α for 24 h) (*p* < 0.05, versus without TNF-α exposure) and increased in the mitochondrial transfer groups (at 24 h after 1 and 25 µg transfer; *p* < 0.05, versus TNF-α exposure without mitochondrial transfer) (Figure 1C). The level of *MMP1*, a collagenase that degrades collagen fibers for matrix regulation [29], was elevated by about 3-fold in damaged tenocytes (*p* < 0.05, versus without TNF-α exposure), and declined after mitochondrial transplantation. All three doses (1, 5, and 25 μg) of mitochondria reduced the expression of *MMP1* to the level observed before TNF-α exposure (*p* < 0.05, versus TNF-α exposure without mitochondrial transfer) (Figure 1D).

Similar results were obtained for TNMD, COL1, and MMP1 protein expression by western blotting. TNMD levels decreased by about 26% upon treatment with TNF-α (*p* < 0.05, versus without TNF-α exposure) and recovered to the pre-damage state upon mitochondrial transfer (1 and 25 µg; *p* < 0.05, versus TNF-α exposure without mitochondrial transfer) (Figure 1E). The expression of COL1, the major component of the tendon matrix, decreased by about 44% compared with that in the no TNF-α exposure group and was upregulated at 24 h after mitochondrial transplantation (1 and 5 μg; *p* < 0.05, versus TNF-α exposure without mitochondrial transfer) (Figure 1F). The MMP1 level that was increased by about 5-fold at 24 h after exposure to 10 ng/mL TNF-α was decreased upon mitochondrial transfer by 65.9 ± 5.2% for 1 µg, 68.6 ± 3.6% for 5 µg, and 67.8 ± 2.7% for 25 µg (*p* < 0.05, versus TNF-α exposure without mitochondrial transfer) (Figure 1G). We also used ruptured mitochondria to confirm the importance of transplanting functional, healthy mitochondria by measuring collagen production in tenocytes. Ruptured mitochondria (1, 5, and 25 µg) did not elicit an increase in collagen content like intact mitochondria did (1 and 5 µg; *p* < 0.05, versus TNF-α exposure without mitochondrial transfer as Figure 1F) (Supplementary Figure S6A).

### 3.3. Mitochondrial Transplantation Attenuated Intracellular Oxidative Stress in TNF-α-Treated Tenocytes In Vitro

**Figure 2:**
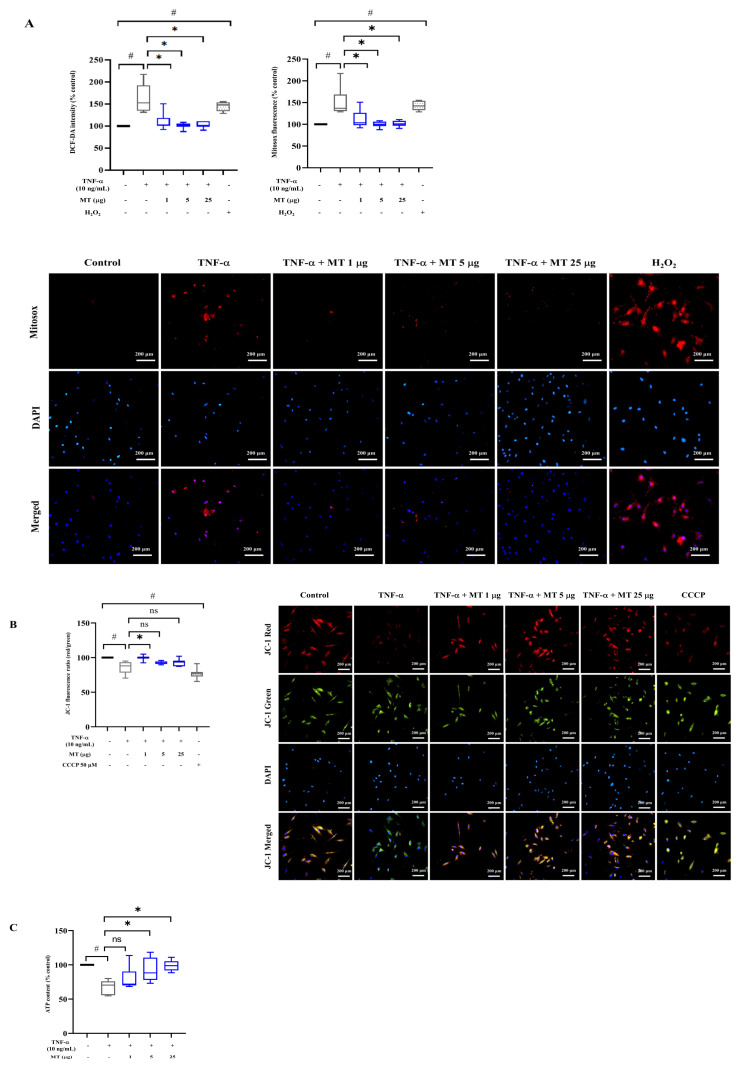

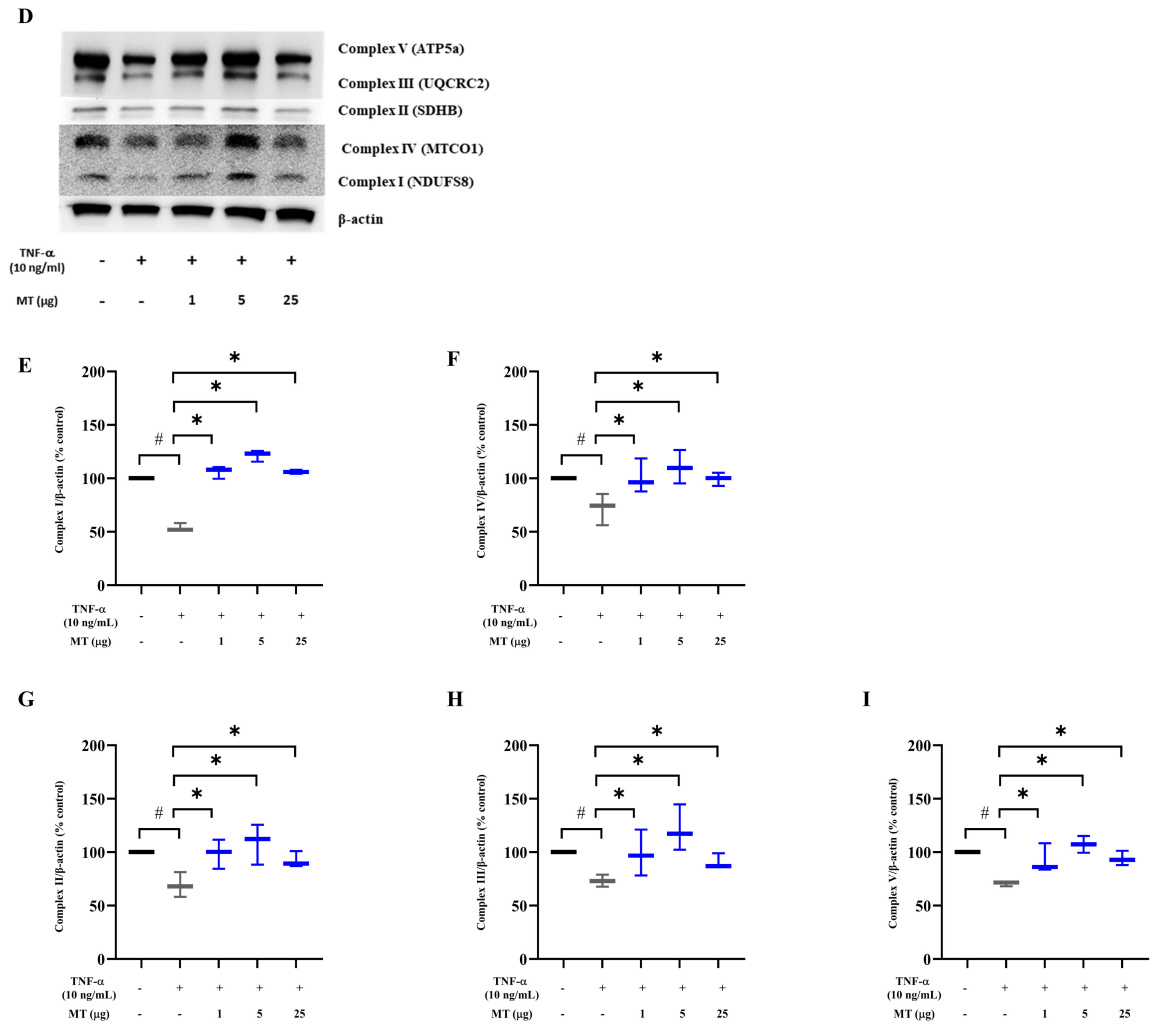
Mitochondrial transplantation reduces elevated oxidative stress, restoring electron transport chain complexes. (**A**–**C**) Effect on oxidative stress and ATP production: changes in (**A**) intracellular ROS (H_2_O_2_: positive control group inducing intracellular oxidative stress); (**B**) MMP (CCCP: positive control group as mitochondrial oxidative phosphorylation uncoupler), and (**C**) ATP production. (**D**–**I**) Effect on mitochondrial OXPHOS complex proteins: (**D**) representative western blots of mitochondrial OXPHOS complex proteins and (**E**–**I**) densitometric quantification of each complex: (**E**) complex I (NDUFS8), (**F**) complex IV (MTCO1), (**G**) complex II (SDHB), (**H**) complex III (UQCRC2), and (**I**) Complex V (ATP5a) (β-actin as loading control). Data represent mean ± standard deviation (SD) (*n* = 3). # *p* < 0.05 between TNF-α (+) group (black) and TNF-α (−) group as control (white), * *p* < 0.05 between MT (+) group damaged by TNF-α (gray) and MT (−) group damaged by TNF-α (black) TNF-α, tumor necrosis factor-α; MT, mitochondria; DCF-DA, dichlorodihydrofluorescein diacetate; H_2_O_2_, hydrogen peroxide; ROS, reactive oxygen species; MMP, mitochondrial membrane potential; CCCP, carbonyl cyanide m-chlorophenyl hydrazone; ATP, adenosine triphosphate; OXPHOS, oxidative phosphorylation; NDUFS8, NADH ubiquinone oxidoreductase core subunit S8; MTCO1, mitochondrially encoded cytochrome C oxidase 1; SDHB, succinate dehydrogenase complex iron-sulfur subunit B; UQCRC2, ubiquinol-cytochrome c reductase core protein 2; ATP5a, ATP synthase F1 subunit alpha.

Approximately 1.4- and 1.5-fold increases in cellular (dichlorodihydrofluorescein diacetate, DCF-DA) and mitochondrial (MitoSOX) ROS levels induced by TNF-α declined upon mitochondrial transplantation in a concentration-dependent manner as follows: for cellular ROS, 19.8%, 25.0%, and 32.6% and for mitochondrial ROS, 26.0%, 33.6%, and 32.5% (1, 5, and 25 µg, respectively; *p* < 0.05, versus TNF-α exposure without mitochondrial transfer) (Figure 2A). The highest dose of mitochondria, 25 µg per 10^5^ cells, almost restored ROS levels to those before the damage. Ruptured mitochondria did not show any changes in ROS and ATP production (Supplementary Figure S6B,C).

We also measured mitochondrial membrane potential after mitochondrial delivery. The TNF-α-treated group showed, approximately, a 13.5% reduction in mitochondrial membrane potential (*p* < 0.05, versus without TNF-α exposure). However, the membrane potential increased to that in the pre-damage state in the mitochondrial transfer group (15.9% for 1 µg, *p* < 0.05, versus TNF-α exposure without mitochondrial transfer) (Figure 2B). Additionally, we evaluated mitochondrial dehydrogenase activity in living cells to measure cell viability [30]. Formazan production decreased by about 40% in damaged tenocytes treated with TNF-α compared with that in the control group without TNF-α exposure. In contrast, the mitochondrial transfer groups showed time-dependent increases in mitochondrial dehydrogenase activity (158.2% for 1 µg, 152.6% for 5 µg, and 201.2% for 25 µg for 24 h of mitochondrial transplantation; *p* < 0.05, versus TNF-α exposure without mitochondrial transfer) (Supplementary Figure S6D, Intact MT). However, the reduction in mitochondrial dehydrogenase activity by TNF-α was not attenuated in the case of ruptured mitochondria (Supplementary Figure S6D, Ruptured MT).

We further found that ATP content was 33% lower in the TNF-α-treated group for 24 h than in the pre-damage state (*p* < 0.05). The amount of intracellular ATP was mostly restored by exogenous mitochondria in a dose-dependent manner (5 and 25 µg; *p* < 0.05, versus TNF-α exposure without mitochondrial transfer) (Figure 2C).

The mitochondrial membrane complex consists of the electron transport chain (ETC) (complexes I to V) and is indispensable for oxidative phosphorylation (OXPHOS) and ATP production [31,32]. Protein levels of all five ETC complexes were suppressed by TNF-α treatment and were restored in the mitochondrial transfer groups (1, 5, and 25 µg) comparable to those in the pre-damage states (Figure 2D–I).

### 3.4. Mitochondrial Transplantation Downregulated Fission Factors and Upregulated Fusion Factors in TNFα-Treated Tenocytes In Vitro

**Figure 3:**
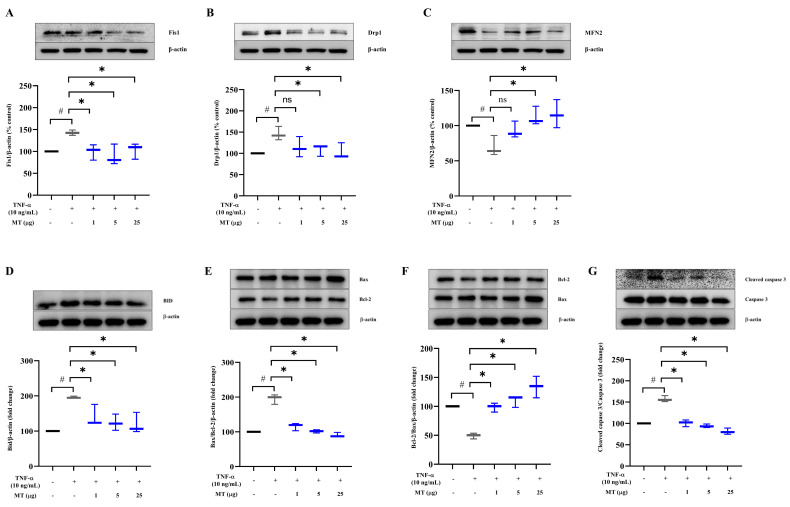

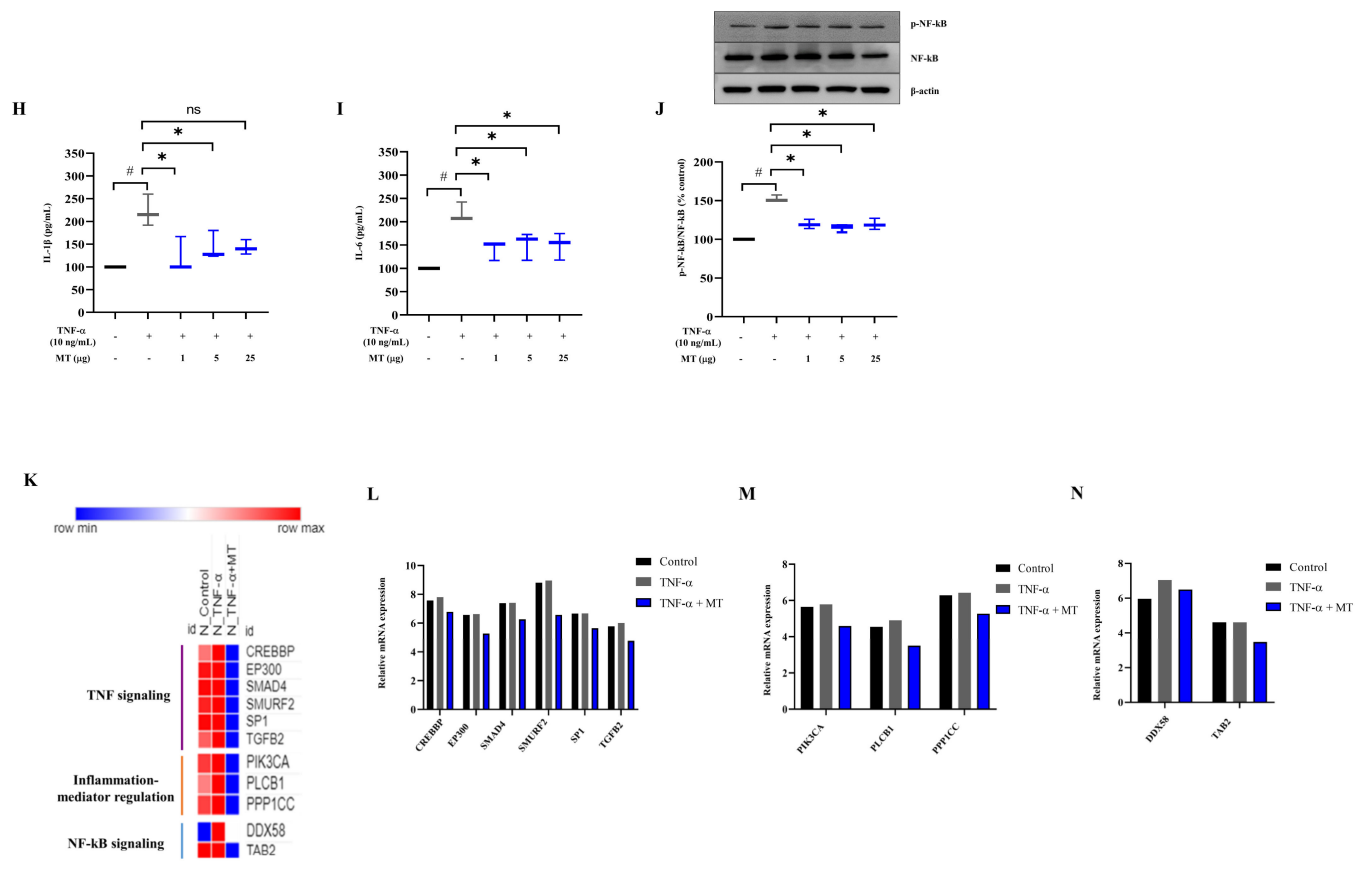
Effect of mitochondrial transplantation on mitochondrial dynamics and apoptosis- and inflammation-related marker levels in TNFα-treated tenocytes in vitro. (**A**–**G**) Effect on mitochondrial dynamics and apoptosis: (**A**–**C**) changes in levels of mitochondrial fission markers, such as Fis1 and Drp1 and the fusion marker MFN2. Representative western blots and densitometric quantification of (**A**) Fis1, (**B**) Drp1, and (**C**) MFN2. (**D**–**G**) Impact on apoptosis-related markers BID, Bax, Bcl-2, and cleaved caspase 3. Representative western blots and densitometric quantification of (**D**) BID, (**E**) Bax, (**F**) Bcl-2, and (**G**) cleaved caspase 3. (**H**–**N**) Effect on inflammation-related markers: (**H**,**I**) show that mitochondrial transplantation decreased the levels of inflammatory markers, such as (**H**) IL-1β and (**I**) IL-6, as determined using ELISA. Representative western blot and densitometric quantification of (**J**) NF-κB signaling. (**A**–**G**), (**J**): β-actin as a loading control. Data represent mean ± standard deviation (SD) (*n* = 3). # *p* < 0.05 between TNF-α (+) group (black) and TNF-α (−) group as control (white), * *p* < 0.05 between MT (+) group damaged by TNF-α (gray) and MT (−) group damaged by TNF-α (black); (**K**) microarray profiling (heat map and hierarchical clustering of differential expression of mRNAs) shows the gene pathways of TNF signaling, inflammation, and NF-κB signaling altered by mitochondrial transplantation in MT (+) group damaged by TNF-α (N_TNF-α + MT) and MT (−) group damaged by TNF-α (N_TNF-α). Blue color in N_TNF-α + MT indicates reduced TNF signaling levels, inflammation mediator regulation, and NF-κB signaling after MT transplantation. The color scale at the top illustrates the relative expression level of mRNA across all samples; (**L**–**N**) shows the relative expressions of mRNA regarding (**L**) TNF, (**M**) inflammation, and (**N**) NF-κB signaling pathways among three groups such as TNF-α with MT, TNF-α without MT, and control. TNF-α, tumor necrosis factor-α; MT, mitochondria; Fis1, Fission 1; Drp1, Dynamin-related protein 1; MFN2, mitofusin 2; BID, BH3-interacting domain death agonist; Bax, Bcl-2 associated X; Bcl-2, B-cell lymphoma 2; IL, interleukin; NF-κB, nuclear factor-kappa B; ELISA, enzyme-linked immunosorbent assay.

Treatment with 10 ng/mL TNF-α for 24 h increased the levels of mitochondrial fission factors, such as Fis1 and Drp1, by about 1.5 fold, whereas fusion-related mitofusin 2 (MFN2) level decreased by about 39% (*p* < 0.05, versus without TNF-α exposure) (Figure 3A–C). Compared with that in the TNF-α-treated group, Fis1 level was suppressed by 43 ± 8.9%, 53 ± 11.8%, and 39 ± 9.1% in groups treated with 1, 5, and 25 µg of mitochondria, respectively (*p* < 0.05, versus TNF-α exposure without mitochondrial transfer) (Figure 3A). After mitochondrial transplantation, the increased Drp1 level was reduced by 25.2 ± 1.4%, and 29.0 ± 1.9% in the groups treated with 5, and 25 µg of mitochondria, respectively (Figure 3B). As a fusion factor, the level of MFN2 increased in a concentration-dependent manner (5 and 25 µg), from the suppressed state, in the presence of TNF-α (Figure 3C). The levels of Drp1, a mitochondrial fission marker, and pNF-κB were synergistically reduced by co-treatment with a mitochondrial division inhibitor (Mdivi) and mitochondria (25 µg) compared with their increased state in the presence of TNF-α (Supplementary Figure S7A,B).

### 3.5. Mitochondrial Transplantation Inhibited Apoptosis in TNFα-Treated Tenocytes In Vitro

Tenocytes exposed to TNF-α for 24 h showed higher levels of pro-apoptotic markers and lower levels of anti-apoptotic proteins than the control group without exposure to TNF-α. At 24 h after the transfer of isolated mitochondria, the damage-induced increase in the level of BID, a pro-apoptotic marker, was decreased at mitochondrial transfer doses of 1, 5, and 25 μg (28.1 ± 3.0%, 36.6 ± 2.3%, and 39.0 ± 3.0%, respectively) (*p* < 0.05, versus TNF-α exposure without mitochondrial transfer) (Figure 3D). Mitochondrial transfer decreased Bax (pro-apoptotic marker; 40.8 ± 1.1%, 48.0 ± 0.5%, and 53.6 ± 0.7%) and increased Bcl-2 (anti-apoptotic marker; 100.6 ± 0.8%, 124.0 ± 1.0%, and 172.5 ± 1.9%) levels at doses of 1, 5, and 25 μg, respectively (*p* < 0.05, versus TNF-α exposure without mitochondrial transfer) (Figure 3E,F). Moreover, activated cleaved caspase 3 level was suppressed by mitochondrial transfer (35.7 ± 0.8%, 40.2 ± 0.4%, and 48.4 ± 0.7% at doses of 1, 5, and 25 μg, respectively) (*p* < 0.05, versus TNF-α exposure without mitochondrial transfer) (Figure 3G). BID and Bcl-2 levels showed qualitatively similar differences between groups subjected to mitochondrial transfer and Mdivi treatment, without synergistic effects (Supplementary Figure S7C,D).

### 3.6. Mitochondrial Transplantation Inhibited Inflammatory Marker Expression in TNFα-Treated Tenocytes In Vitro

Levels of the pro-inflammatory cytokines IL-1β and IL-6 in tenocytes increased in response to TNF-α (by about 2-fold) compared with those in the control group not subjected to TNF-α (*p* < 0.05). Mitochondrial transfer suppressed IL-1β (90 ± 9.6% and 55 ± 10.0% at doses of 1 and 5 μg, respectively) (*p* < 0.05, versus TNF-α exposure without mitochondrial transfer) (Figure 3H) and IL-6 (78 ± 6.1%, 68 ± 8.7%, 69 ± 8.5% at doses of 1, 5, and 25 μg, respectively) (*p* < 0.05, versus TNF-α exposure without mitochondrial transfer) expression (Figure 3I). Mitochondrial transplantation (1, 5, and 25 μg) also suppressed (21.6 ± 6.0%, 24.9 ± 4.9%, and 21.6 ± 7.2% at doses of 1, 5 and 25 μg, respectively (*p* < 0.05, versus TNF-α exposure without mitochondrial transfer) the increased phosphorylation of NF-κB (approximately 1.5-fold upon TNF-α exposure), a key pathway in the inflammatory cascade (Figure 3J). We next performed supervised hierarchical clustering (HC) of differentially expressed genes (with statistical cut off level of *p* < 0.05) in each group and created heatmaps to illustrate the expression patterns for TNF-α exposure and TNF-α exposure with mitochondrial transfer (Figure 3K–N and Supplementary Figure S8). In this study, such inflammatory and NF-κB signaling changes were verified in a comparative microarray analysis of three groups (an intact control group and two groups of cells treated with TNF-α alone and TNF-α + mitochondria 25 μg, respectively).

### 3.7. Mitochondrial Transplantation Alleviated Collagenase-Induced Tendinopathy in Rats In Vivo

**Figure 4:**
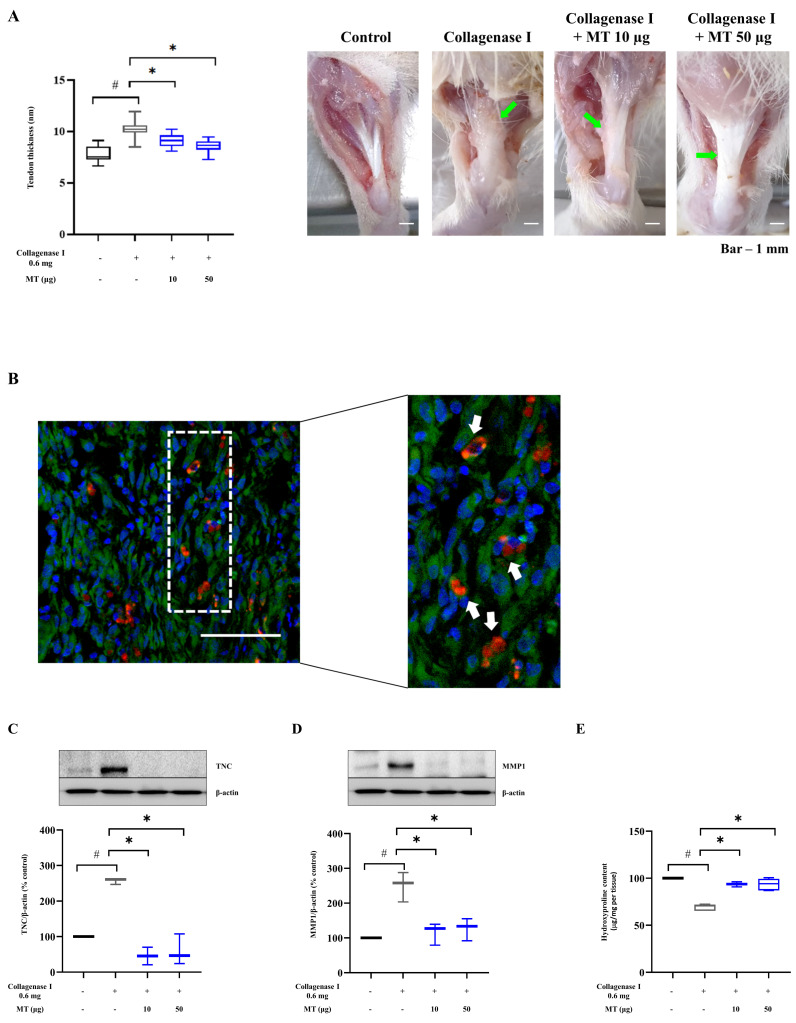
Exogenous mitochondria incorporated into Achilles tendon reduce tendon swelling and elevated TNC/MMP1 level, enhancing collagen production in the collagenase-induced tendinopathy model. Mitochondria (10 and 50 μg) were locally injected into Achilles tendons at 2 weeks after collagenase-induced injury. (**A**) Changes in hindlimb thickness and tendon tissue gross appearance before and after mitochondrial injection; (**B**) confocal imaging of tendon tissue after mitochondrial injection: red and green colors indicate exogenous mitochondria from L6 rat myoblast (MitoTracker CMSR_OS_ red, white arrows) and endogenous mitochondria of harvested tendon tissue (MitoTracker Green), respectively, at 2 weeks after collagenase injection with DAPI nuclear staining (blue) (scale bar, 400 μm); representative western blots and densitometric quantification of (**C**) TNC and (**D**) MMP1 (β-actin as loading control); (**E**) hydroxyproline assay for COL1 data represent mean ± standard deviation (SD) (*n* = 3). # *p* < 0.05 between collagenase (+) group (black) and collagenase (−) group as control (white), * *p* < 0.05 between MT (+) group damaged by collagenase (gray) and MT (−) group damaged by collagenase (black). MT, mitochondria; TNC, Tenascin C; MMP1, matrix metalloproteinase 1; COL, collagen.

A rat model of tendinopathy was prepared by collagenase injection into the Achilles tendon [33]. The tendon thickness increased significantly at 1 and 2 weeks after 0.6 mg collagenase injection compared with that in the control group (*p* < 0.05). Swelling subsided at 1 and 2 weeks after local injection of 10 and 50 μg mitochondria into Achilles tendons (*p* < 0.05, versus collagenase injection without mitochondrial transfer) (normal control (rat number = 3, Achilles tendon number = 6), positive control injured by collagenase (rat number = 3, Achilles tendon number = 6), low mitochondrial concentration 10 μg (rat number = 3, Achilles tendon number = 6), and higher concentration 50 μg (rat number = 3, Achilles tendon number = 6) (Figure 4A and Supplementary Figure S9A).

We evaluated whether injected exogenous mitochondria were incorporated into Achilles tendon tissues. Mitochondria stained with CMXRos were administered to the Achilles tendon. After 2 weeks, the tendon tissues were harvested, and CMXRos signals were detected by confocal microscopy (Figure 4B).

Furthermore, protein levels of Tenascin C (TNC) and MMP1 were measured. TNC is involved in the tendon tissue repair process, which modulates MMP expression and whose level increases under pathological conditions. TNC level increased approximately 2.6 folds in the injured group (versus normal control group) and decreased by 82.3 ± 2.5% and 76.8 ± 4.3% in the groups treated with 10 and 50 μg of mitochondria, respectively (*p* < 0.05, versus collagenase-injected tissue without mitochondrial transfer) (Figure 4C). The level of MMP1, a substrate-degrading enzyme, increased by about 2.5 fold in the injured group compared with that in the normal control group and was suppressed by 53.9 ± 3.2% and 49.2 ± 3.2% in the 10 and 50 μg mitochondrial transfer groups, respectively (*p* < 0.05, versus collagenase-injected tissue without mitochondrial transfer) (Figure 4D). Collagen content was reduced by 32 ± 0.4% after collagenase injection compared with that in the normal control group and increased by about 25% following 10 and 50 μg mitochondrial injection (*p* < 0.05, versus collagenase-injected tissue without mitochondrial transfer) (Figure 4E).

### 3.8. Mitochondrial Transplantation Promoted Mitochondrial Dynamics and Inhibited Apoptosis and Inflammation in Collagenase-Induced Tendinopathy in Rats In Vivo

**Figure 5:**
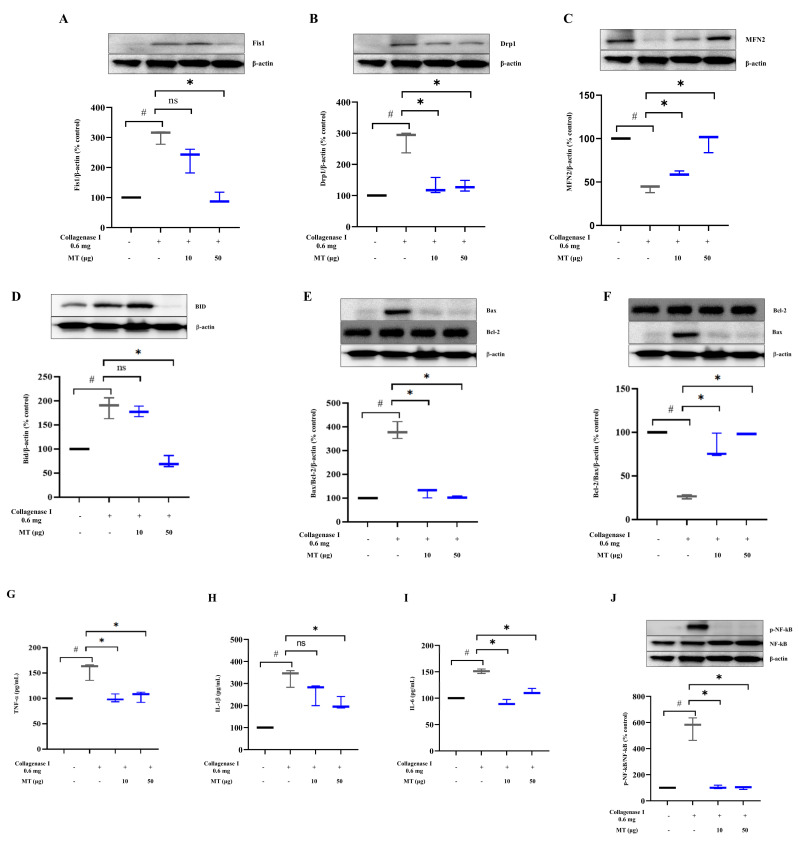
Effect of mitochondrial transplantation on mitochondrial dynamics and apoptosis- and inflammation-related marker levels in collagenase-induced tendinopathy in rats in vivo. (**A**–**C**): changes in levels of mitochondrial fission markers such as Fis1 and Drp1 and the fusion marker MFN2. Representative western blots and densitometric quantification of (**A**) Fis1, (**B**) Drp1, and (**C**) MFN2; (**D**–**F**): Effects on apoptosis-related markers BID, Bax, and Bcl-2. Representative western blots and densitometric quantification of (**D**) BID, (**E**) Bax, and (**F**) Bcl-2; third-row panel (**G**–**I**) shows that mitochondrial transplantation decreased the levels of inflammatory markers such as (**G**) TNF-α, (**H**) IL-1β, and (**I**) IL-6, as determined using ELISA. Representative western blot and densitometric quantification of (**J**) NF-κB signaling; (**A**–**F**) and (**J**): β-actin as a loading control. Data represent mean ± standard deviation (SD) (*n* = 3). # *p* < 0.05 between collagenase (+) group (black) and collagenase (−) group as control (white), * *p* < 0.05 between MT (+) group damaged by collagenase (gray) and MT (−) group damaged by collagenase (black) MT, mitochondria; Fis1, Fission 1; Drp1, dynamin-related protein 1; MFN2, mitofusin 2; BID, BH3-interacting domain death agonist; Bax, Bcl-2 associated X; Bcl-2, B-cell lymphoma 2; TNF-α, tumor necrosis factor-α; IL, interleukin; NF-κB, nuclear factor-kappa B; ELISA, enzyme-linked immunosorbent assay.

To determine the impact of mitochondrial transplantation on mitochondrial dynamics, protein levels of Fis1, Drp1, and MFN2 were analyzed. In collagenase-injected tissues, Fis1 and Drp1 levels increased by about 3-fold each, and MFN2 level decreased by about 55% compared with those in the pre-damage group (*p* < 0.05). After the delivery of 50 µg mitochondria by local injection, Fis1 level was reduced (*p* < 0.05, versus collagenase-injected tissue without mitochondrial transfer) (Figure 5A). Drp1 level was dramatically suppressed by about 53.8 ± 2.6% and 53.0 ± 1.7% by mitochondrial transplantation (10 and 50 µg) compared with that in collagenase-injected tissue (*p* < 0.05) (Figure 5B). MFN2 level increased by 34.8 ± 0.2% and 106.4 ± 1.0% after the injection of mitochondria at doses of 10 and 50 μg, respectively, from the suppressed state, upon collagenase treatment (*p* < 0.05, versus collagenase-injected tissue without mitochondrial transfer) (Figure 5C).

Next, apoptotic proteins (BID, Bax, and Bcl-2) were assessed at 2 weeks after mitochondrial transplantation. Collagenase I-induced increases in BID (by approximately 1.9-fold) and Bax (approximately 3.5-fold) levels declined upon mitochondrial transplantation (50 μg for BID, 10 and 50 μg for Bax) (*p* < 0.05, versus collagenase-injected tissue without mitochondrial transfer) (Figure 5D,E). The decreased Bcl-2 level (by 73.7% at 2 weeks after collagenase injection [*p* < 0.05, versus normal control group]) returned to the control level upon 10 and 50 μg mitochondrial transplantation (*p* < 0.05, versus collagenase-injected tissue without mitochondrial transfer) (Figure 5F).

Inflammatory cytokines, such as TNF-α, IL-1β, and IL-6, were also analyzed. Compared with that in the control group, IL-1β level increased by about 3.3-fold, and TNF-α and IL-6 levels increased by about 1.5-fold each (Figure 5G–I). When mitochondria were injected into the collagenase-damaged Achilles tendons, TNF-α returned to the control level, regardless of the dose of mitochondria (10 and 50 μg) (*p* < 0.05, versus collagenase-injected tissue without mitochondrial transfer) (Figure 5G). IL-1β level decreased compared with that in the damaged tissue (50 μg mitochondrial transplantation, 36.6%) (*p* < 0.05, versus collagenase-injected tissue without mitochondrial transfer) (Figure 5H). Besides, IL-6 level was suppressed by 37.4% and 23.4% in the 10 and 50 μg mitochondrial transfer groups, respectively (*p* < 0.05, versus collagenase-injected tissue without mitochondrial transfer) (Figure 5I). Furthermore, in the collagenase-injected group, NF-κB pathway expression increased by about 5.6-fold, and, in the mitochondrial transfer groups, the phosphorylation of NF-κB was abruptly suppressed by about 81.4% and 82.4% in the 10 and 50 μg of mitochondrial transfer groups, respectively (*p* < 0.05, versus collagenase-injected tissue without mitochondrial transfer) (Figure 5J).

## 4. Discussion

This study showed that intact exogenous mitochondrial transplantation could have protective effects against TNF-α-induced damage in tenocytes and a collagenase-induced animal model of tendinopathy. Inflammation is the primary pathogenic mechanism of tendinopathy [9,34], and mitochondrial transplantation suppressed NF-κB signaling along with pro-inflammatory marker (IL-1β and IL-6) levels.

Tendinopathy is characterized as “failed healing” which refers to the incomplete recovery of mechanical strength and inferior tissue quality mainly due to tendon tissue avascularity [35,36]. Such poor blood supply and hypoxic stress result in disturbed mitochondrial homeostasis, inducing inflammation or apoptosis in tendinopathy [14,16]. Mitochondrial dysfunction leads to amplification of inflammation via uncontrolled release of ROS and NF-κB activation, which comprise a redox-sensitive inflammatory pathway [37,38,39]. A crosstalk between NF-κB signaling and ROS production has been reported [17]. Injury and stress cause the release of mitochondrial ROS as well as damage-associated molecular patterns, such as mtDNA, which can result in cell death and activate the immune responses [40,41]. Our results showed that the damaged-induced increase in the level of NF-κB, a critical regulator of tissue inflammation, was suppressed by mitochondrial transplantation.

Mitochondria were successfully incorporated into tenocytes using centrifugation and revitalized damaged tenocytes. TNMD, COL1, MMP1, and TNC levels were altered upon mitochondrial transplantation, along with reductions in pro-inflammatory marker levels. TNMD is a specific tenocyte-related marker [27]. The knockout of TNMD results in tenocyte senescence and collagen disruption [42,43]. COLI is the main component of the tendon matrix (~95%) and is involved in the transduction of high mechanical forces and production of collagen fibrils by tenocytes [44]. MMP1 is an MMP subtype that resides in fibroblasts with ECM degradation [45]. MMP1 expression increases in damaged tenocytes and torn human tendons [35,46]. TNC is associated with the tendon response to compression [47]. These changes are accompanied by reduced inflammation and apoptosis upon the modulation of mitochondrial dynamics. Apoptosis induced by mitochondrial dysfunction, as determined by assessing BID, Bax, Bcl-2, and caspase 3 signaling, was alleviated upon the recovery of tendon-related marker levels.

Exogenous mitochondria might contribute to or replace the activity of endogenous mitochondria. Mitochondrial function can be measured by changes in ROS and ATP levels and mitochondria-membrane potential [48]. In particular, the proper balance between ROS and ATP plays a pivotal role in mitochondrial functions. We observed mitochondrial functional recovery upon the delivery of mitochondria to damaged cells. The delivery of ruptured mitochondria did not affect intracellular mitochondrial functions. These results indicate that viable and healthy mitochondria are critical for inducing beneficial mitochondrial effects on collagen production, oxidative stress, ATP content, and cell viability.

When mitochondria fail to function properly, the equilibrium between mitochondrial fusion and fission shifts [49], causing excessive ROS generation, leading to NLRP3 inflammasome activation and NF-κB phosphorylation. We expect mitochondrial dynamics to be impacted by exogenous mitochondria. In general, mitochondrial fission is activated upon injury, and mitochondrial fission and fusion pathways interact [50]. When exogenous mitochondria were transferred to injured tenocytes, the level of injury-induced Fis1 decreased, and fusion levels increased. We hypothesized that excessive mitochondrial fission contributes to inflammation-induced tendinopathy. Further corroborating this finding, we observed that fission and inflammation were altered by treatment with the fission inhibitor Mdivi-1 in addition to mitochondrial transfer. The combination of fission inhibition and mitochondrial delivery resulted in a more significant reduction in fission and inflammation marker levels than each treatment alone.

Mitochondria are a major source of ROS under physiological conditions; ROS mediate redox signaling [17]. Excessive mitochondrial ROS causes cell injury linked to various processes, from aging to chronic diseases, such as neurodegeneration and cancers [51]. Mitochondrial ROS induce pro-inflammatory cytokine production, and a complex interplay underlies ROS and NF-κB pathway interactions [17]. The anti-inflammatory effect of mitochondrial transfer suggests a promising therapeutic strategy because inflammation is a fundamental mechanism of tendinopathy pathogenesis [9].

The delicate balance between fission and fusion determines mitochondrial network morphology and affects mitochondrial functions [49]. Mitochondrial fragmentation is necessary for ROS production [52] and cell death [53,54]. Thus, aberrant mitochondrial dynamics and excessive ROS generate a vicious cycle leading to cell death and inflammation [55]. Our findings were consistent with those of previous studies showing that TNF-α increases mitochondrial ROS formation and Drp1 expression, resulting in mitochondrial fragmentation [56].

These findings were validated by microarray data related to TNF signaling, apoptosis, calcium signaling, inflammation-mediator regulation, and other processes. Significantly affected signaling pathways in this analysis correspond to those reportedly affected in previous cell and animal studies.

This study had several limitations. First, we could not obtain consistency between the dose-dependent effects of delivered mitochondrial doses (1, 5, and 25 µg per 1 × 10^5^ tenocytes) and outcome variables. This might be attributed to the ceiling effect of higher concentrations of mitochondria. Various molecules released from mitochondria (mitochondrial DAMPs, damage-associated molecular patterns) can induce pro-inflammatory properties and have been implicated in pathologic conditions [57]. Higher mitochondrial doses might be associated with more mitochondrial DAMPs, which could counteract mitochondria transplantation’s favorable effects. Further studies shall determine the proper dose of mitochondria within a lower range. Second, we used different delivery methods for exogenous mitochondria in tenocytes and rat Achilles tendons. Simple co-incubation with mitochondria could be a more appropriate strategy for cell-based experiments, considering the distribution of injected mitochondria in the rat Achilles tendons. The co-incubation of mitochondria with tendon cells is also an intracellular mitochondrial delivery method [58]. Centrifugation, as an alternative to co-incubation, has a high transfer efficiency and reproducibility [26]. Third, tenocyte-related marker levels were inconsistent between in vitro (TNMD) and in vivo (TNC) experiments. TNMD is a well-known tenocyte-specific marker, and its level increases upon exposure to excessive stress. The differences in results between in vitro and in vivo experiments might be due to the difference in materials used to damage cells or tissues (TNF-α versus collagenase) and in delivery methods (centrifugation versus co-incubation). Other markers showed changes consistent with those in common anti-inflammatory and anti-apoptosis mechanisms. Alternatively, microRNAs could be a potential biomarker of tendon pathologies in follow-up studies [59]. Fourth, in the animal study, the biomechanical strength of tendon tissues and behavioral outcomes, including the pain response and gait, were not evaluated. Last, DCF-DA was used to quantify the cellular redox environment. Although DCF-DA is a mostly used probe to detect ROS, it lacks reaction with several oxidant species, such as H2O2 [60]. Newer probes could be used in future studies.

## 5. Conclusions

This study provides the first evidence for the therapeutic effects of mitochondrial transfer in tendinopathy. Exogenous mitochondria were successfully delivered into damaged tenocytes and tendons. They modulated inflammation and apoptosis and recovered the collagen component of the extracellular matrix. Exogenous mitochondrial transfer may become a novel approach for the treatment of tendinopathy.

## Data Availability

The data are available from the corresponding authors upon reasonable request.

## Supplementary Materials

The following are available online at https://www.mdpi.com/article/10.3390/antiox10050696/s1, Figure S1: Establishment of in vitro tendinopathy model by exposure to TNF-α. Figure S2: Isolation of mitochondria and mitochondrial labelling, Figure S3: Full-length image of western blot analysis, Figure S4: Characterization of isolated mitochondria from human umbilical cord mesenchymal stem cells (UC-MSCs), Figure S5: Full fluorescence microscopy of tenocytes after mitochondria transfer, Figure S6: Ruptured mitochondria do not show beneficial effects in contrast to intact mitochondria in TNFα-treated tenocytes in vitro, Figure S7: Mitochondrial transplantation and mitochondrial division inhibitor (Mdivi) synergistically reduce Drp1, a fission marker, and NF-κB signaling, Figure S8: Microarray profiling of mRNAs expression on tenocytes, Figure S9: Swollen tendon after collagenase injection decreased by mitochondrial transplantation in a rat model of tendinopathy, Table S1: The primers used for qPCR.

## Abbreviations

ATP	adenosine triphosphate
ATP5a	ATP synthase F1 subunit alpha
Bax	Bcl-2 associated X
Bcl-2	B-cell lymphoma 2
BID	BH3-interacting domain death agonist
BSA	bovine serum albumin
CCCP	carbonyl cyanide m-chlorophenyl hydrazone
COL1	collagen 1
DAPI	4′6-diamidino-2-phenylindole
DMAB	dimethylamino benzaldehyde
DCF-DA	dichlorodihydrofluorescein diacetate
DPBS	Dulbecco’s phosphate-buffered saline
DRP1	dynamin-related protein 1
EGTA	ethylene glycol tetra acetic acid
ELISA	enzyme-linked immunosorbent assay
Fis1	fission 1
H_2_O_2_	hydrogen peroxide
HCl	hydrogen chloride
HEPES	hydroxyethyl piperazine ethanesulfonic acid
HRP	horseradish peroxidase
IACUC	Institutional Animal Care and Use Committee
IL-1β	interleukin 1 beta
IL-6	interleukin 6
MFN2	mitofusin 2
MitoTracker CMXRos red	CMXRos red
MMP	mitochondrial membrane potential
MMP1	matrix metalloproteinase-1
MT	mitochondria
MTCO1	mitochondrially encoded cytochrome C oxidase 1
NaOH	sodium hydroxide
NDUFS8	NADH ubiquinone oxidoreductase core subunit S8
NF-κB	nuclear factor kappa-light-chain-enhancer of activated B cells
OXPHOS	oxidative phosphorylation
P/S	penicillin/streptomycin
PBS	phosphate-buffered saline
Polyacrylamide gel electrophoresis	PAGE
PVDF	polyvinylidene difluoride
RIPA	radio immunoprecipitation assay
ROS	reactive oxygen species
SDHB	succinate dehydrogenase complex iron-sulfur subunit B
SDS	sodium dodecyl sulfate
TMB	tetramethylbenzidine
TNC	tenascin C
TNF-α	tumor necrosis factor-α
TNMD	tenomodulin
UC-MSC	Umbilical cord-mesenchymal stem cell
UQCRC2	ubiquinol-cytochrome c reductase core protein 2
