# Supplementary material for: Mitochondrial Transplantation Modulates Inflammation and Apoptosis, Alleviating Tendinopathy Both In Vivo and In Vitro
Source: Antioxidants (Basel). 2021 Apr 28;10(5):696. doi: 10.3390/antiox10050696 (PMC8146308; doi:10.3390/antiox10050696)
Supplement: Supplementary file 1 [file antioxidants-10-00696-s001.zip › antioxidants-1177808-supplementary/Supplementary Figures R2/Supplementary_legends R2.pdf]

Supplementary figure legends

Supplementary figure 1. Establishment of *in vitro* tendinopathy model by exposure to TNF-α.

Tenocytes were treated with TNF-α in a time-and dose-dependent manner. Tenocyte-related markers were compared to normal conditions at gene and protein levels. Also, mitochondrial oxidative stress, ATP and MMP functions, and apoptosis-related markers were used for various conditions. (A) *TNMD*, (B) *COL1* and (C) *MMP1* gene levels with TNF-α 10 ng/mL for 0, 6, 12, 24 hours (normalized to GAPDH) (D) mRNA expressions and (E) protein expressions of 24 hours after TNF-α 10 ng/mL treatment (normalized to GAPDH in gene and β-actin in protein level) (F) Intracellular ROS and (G) ATP contents according to four doses of TNF-α (10, 50, 100, and 500 ng/mL) at 24 hours and for two hours. (H) Mitochondrial membrane potential by TNF-α 10 ng/mL for 24 hours, CCCP: positive control group as mitochondrial oxidative phosphorylation uncoupler (I) BID, (J) Bax and (K) Bcl-2; (L-N) Time course of the mitochondrial apoptosis pathway in response to TNF-α 10 ng/mL; (L) BID, (M) Bax and (N) Bcl-2 (I to N: normalized to β-actin)

Data are mean ±SD (n=3). # $p < 0.05$ between TNF-α (+) group (black bar as 24 h treatment at 10 ng/mL and gray bars as other higher concentrations and shorter treatment durations) and TNF-α (-) group as control (white).

TNF-α, tumor necrosis factor-α; TNMD, tenomodulin; COL, collagen; MMP, matrix metalloproteinase; DCF-DA, dichlorodihydrofluorescein diacetate; ATP, adenosine triphosphate; BID, BH3-interacting domain death agonist; Bax, Bcl-2 associated X; Bcl-2, B-

cell lymphoma 2; ROS, reactive oxygen species; CCCP, carbonyl cyanide m-chlorophenyl hydrazone

Supplementary figure 2. Isolation of mitochondria and mitochondrial labelling

(A) Flow chart of mitochondrial isolation by differential centrifugation

(B) Experimental scheme of mitochondrial labelling by MitoTracker Green for tenocytes and MitoTracker CMXRos red for donor cells

Supplementary figure 3. Full-length image of western blot analysis

Full-length photos of western blot analysis in Fig 1(E–G), 2(D), 3(A–G, J), 4(C and D), 5(A–F, J).

Supplementary figure 4. Characterization of isolated mitochondria from human umbilical cord mesenchymal stem cells (UC-MSCs)

Mitochondria and cytosol fractions of UC-MSCs were divided. Mitochondrial markers (AIF, Tom20, Cox IV, and cytochrome C) were measured of isolated mitochondria. Then, functions of isolated mitochondria were also compared with those in ruptured mitochondria to investigate the roles of structural integrity preservation of isolated mitochondria. Mitochondria were ruptured by the physical method. (A) Characterization of isolated mitochondria; (B-E) comparisons of intact and ruptured mitochondria; (B) size; (C) ATP contents and synthesis; (D) Zeta potential as hydrodynamic size and surface charge; (E) JC-1for mitochondrial membrane

potential

Data are mean ±SD (n=3). # *p*< 0.05 between intact mitochondria and ruptured mitochondria

AIF, apoptosis-inducing factor; Tom, translocase of the outer mitochondrial membrane; COX IV, cytochrome c oxidase subunit 4; PCNA, proliferating cell nuclear antigen; ATP, adenosine triphosphate

Supplementary figure 5. Full fluorescence microscopy of tenocytes after mitochondria transfer

Isolated mitochondria (Mitotracker CMXR$_{OS}$ Red) were transferred into recipient tenocytes (Mitotracker Green). After staining, tenocytes were washed, fixed, and mounted using DAPI mounting medium-labeled nuclei. Original magnification, x200.

TNF-α, tumor necrosis factor-α; MT, mitochondria; DAPI, 4, 6-diamidino-2-phenylindole

Supplementary figure 6. Ruptured mitochondria do not show beneficial effects in contrast to intact mitochondria in TNFα-treated tenocytes *in vitro*.

Ruptured mitochondria were used to study the importance of an intact form of mitochondria for collagen synthesis and controlling oxidative stress, ATP contents, and tenocyte viability. Mitochondria were mechanically ruptured by the physical method. (A) COL1, no change by ruptured MT; (B) DCF-DA, no change by ruptured MT (gray bars); (C) Difference between intact and ruptured MT in ATP contents; (D) Mitochondrial dehydrogenase activity.

MT, mitochondria; TNF-α, tumor necrosis factor-α, DCF-DA, dichlorodihydrofluorescein

diacetate; ATP, adenosine triphosphate

Supplementary figure 7. Mitochondrial transplantation and mitochondrial division inhibitor (Mdivi) synergistically reduce Drp1, a fission marker, and NF-κB signaling.

Representative western blots and densitometric quantification of (A) Drp1, (B) NF-κB, (C) Bax, and (D) Bcl-2. Mitochondrial transplantation reduced (A) Drp1, a fission marker, which was augmented by Mdivi-1 treatment. Such a synergistic effect was shown in (B) NF-κB signaling. However, mitochondria and Mdivi-1 did not provide additional effects in (C) Bax (pro-apoptosis) and (D) Bcl-2 (anti-apoptosis) markers.

Data are mean±SD (n=3). # $p < 0.05$ between TNF-α (+) tenocytes (black) and TNF-α (-) tenocytes (white), * $p < 0.05$ between TNF-α (+) tenocytes with MT and/or Mdivi (gray) and only TNF-α (+) tenocytes without any additional manipulation (black)

Mdivi-1; Mitochondrial division inhibitor. Drp1, dynamin-related protein 1; Bax, Bcl-2 associated X; Bcl-2, B-cell lymphoma 2; NF-κB, nuclear factor-kappa B

Supplementary figure 8. Microarray profiling of mRNAs expression on tenocytes

Microarray analysis was performed with RNA extracted from an intact control group and two cell groups treated with TNF-α alone and TNF-α + mitochondria 25 μg (hierarchical clustering of differential expression of mRNAs: red, up-regulated gene expressions; blue, down-regulated gene expression). Gene pathways: TNF signaling, Apoptosis, Calcium signaling, Inflammation, NF-κB signaling, ECM-receptor interaction, and oxidative phosphorylation

Supplementary figure 9. Swollen tendon after collagenase injection decreased by mitochondrial transplantation in a rat model of tendinopathy.

(A, B) Tendon thickness, (C, D) Bodyweight during two weeks (before mitochondrial transplantation): Tendons were thickened by collagenase injection during two weeks (A) and became thin after mitochondrial transplantation during two weeks (B). There was no meaningful change in body weights (C and D).

(A, C) Date are mean ±SD. # $p < 0.05$ between collagenase (+) group (gray) and collagenase (-) group as control (black),

(B, D) Data are mean ±SD. # $p < 0.05$ between collagenase (+) group (black) and collagenase (-) group as control (white), * $p < 0.05$ between MT (+) group damaged by collagenase (gray) and MT (-) group damaged by collagenase (black)
