# Supplementary figures and images for: Mitochondrial Transplantation Modulates Inflammation and Apoptosis, Alleviating Tendinopathy Both In Vivo and In Vitro
Source: Antioxidants (Basel). 2021 Apr 28;10(5):696. doi: 10.3390/antiox10050696 (PMC8146308; doi:10.3390/antiox10050696)

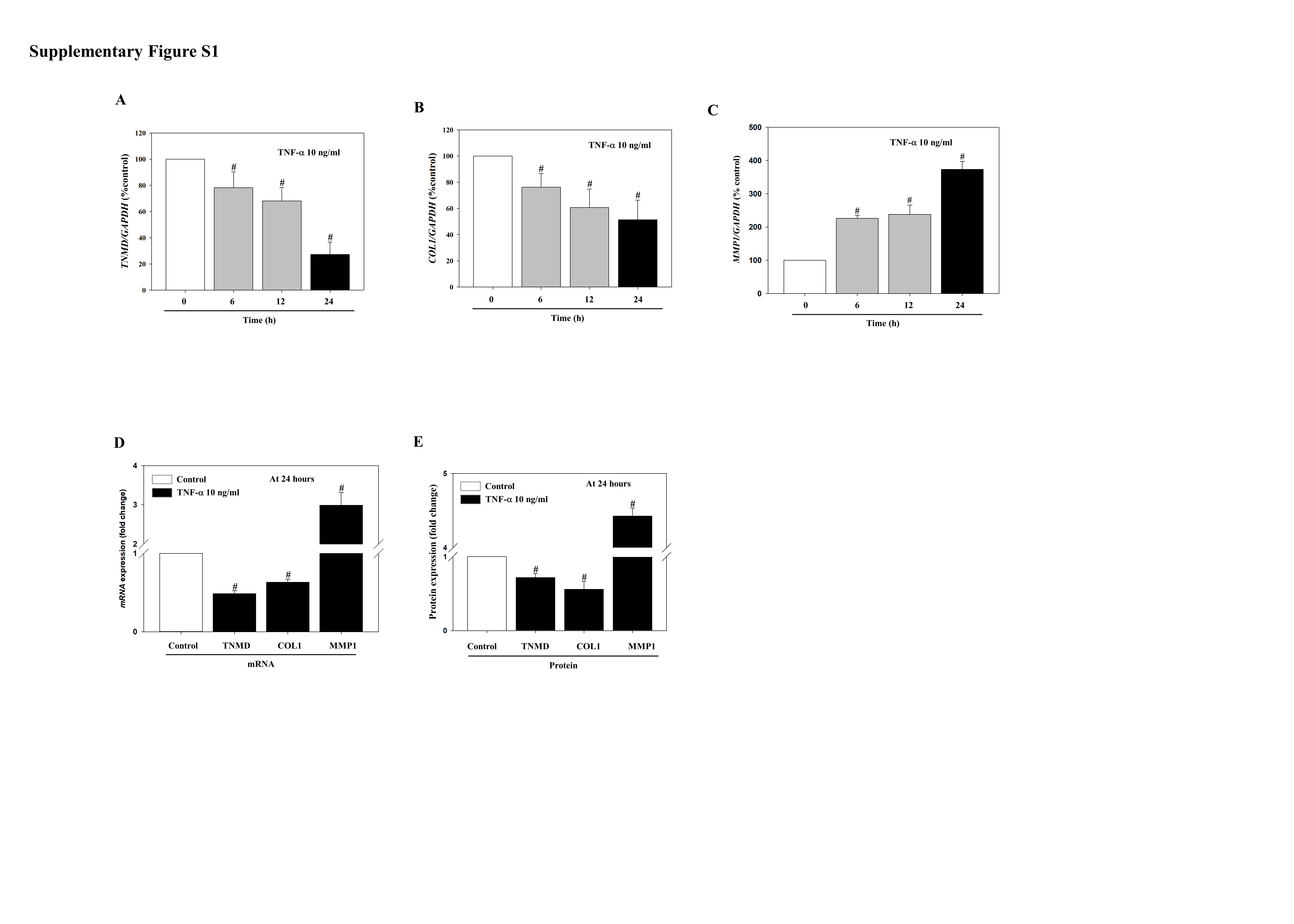

Supplement: Supplementary file 1 [file antioxidants-10-00696-s001.zip › antioxidants-1177808-supplementary/Supplementary Figures R2/Sup_Fig._S1(A-E)_R2.TIF]

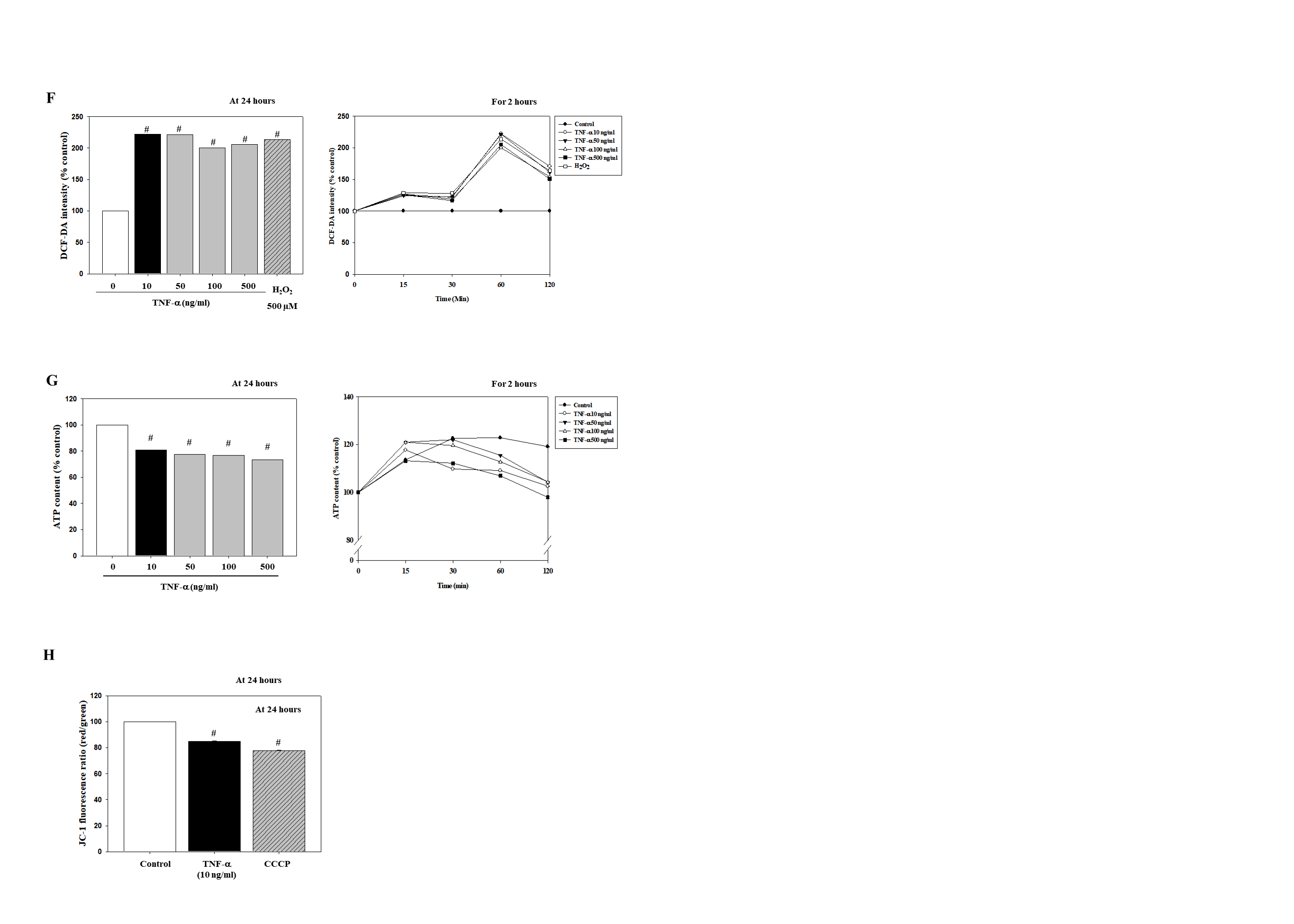

Supplement: Supplementary file 1 [file antioxidants-10-00696-s001.zip › antioxidants-1177808-supplementary/Supplementary Figures R2/Sup_Fig._S1(F-H)_R2.tif]

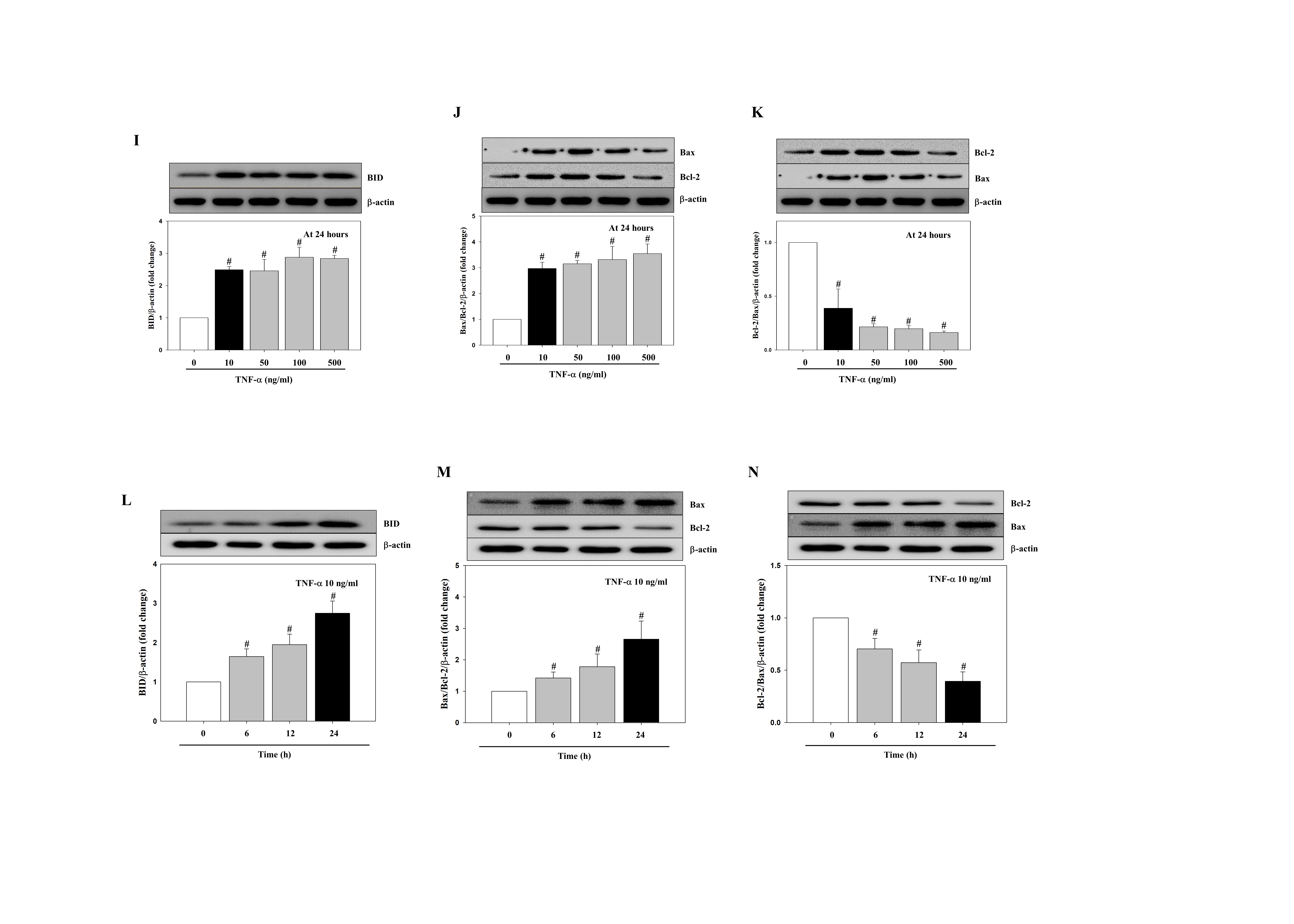

Supplement: Supplementary file 1 [file antioxidants-10-00696-s001.zip › antioxidants-1177808-supplementary/Supplementary Figures R2/Sup_Fig._S1(I-N)_R2.TIF]

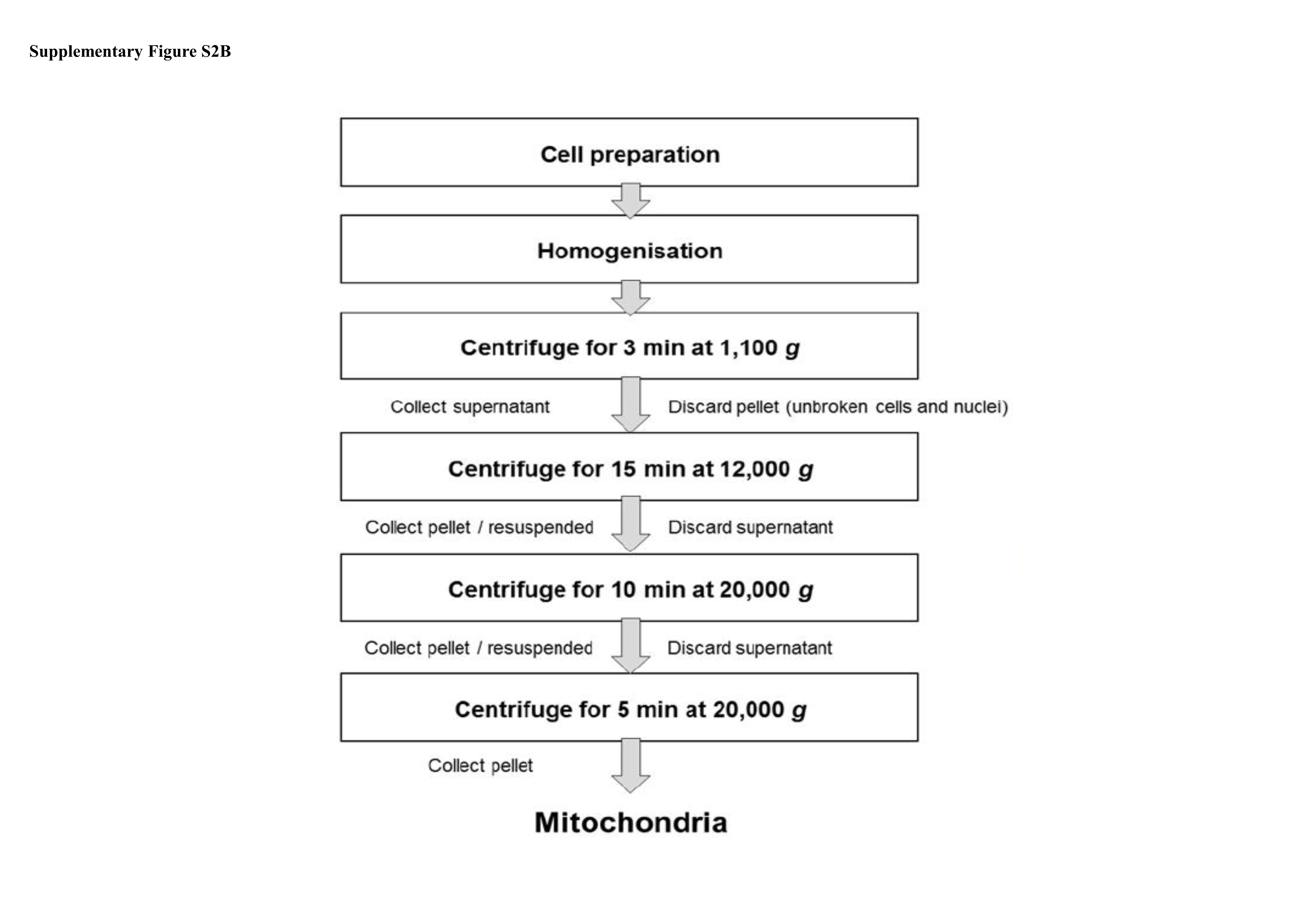

Supplement: Supplementary file 1 [file antioxidants-10-00696-s001.zip › antioxidants-1177808-supplementary/Supplementary Figures R2/Sup_Fig._S2(A)_R2.TIF]

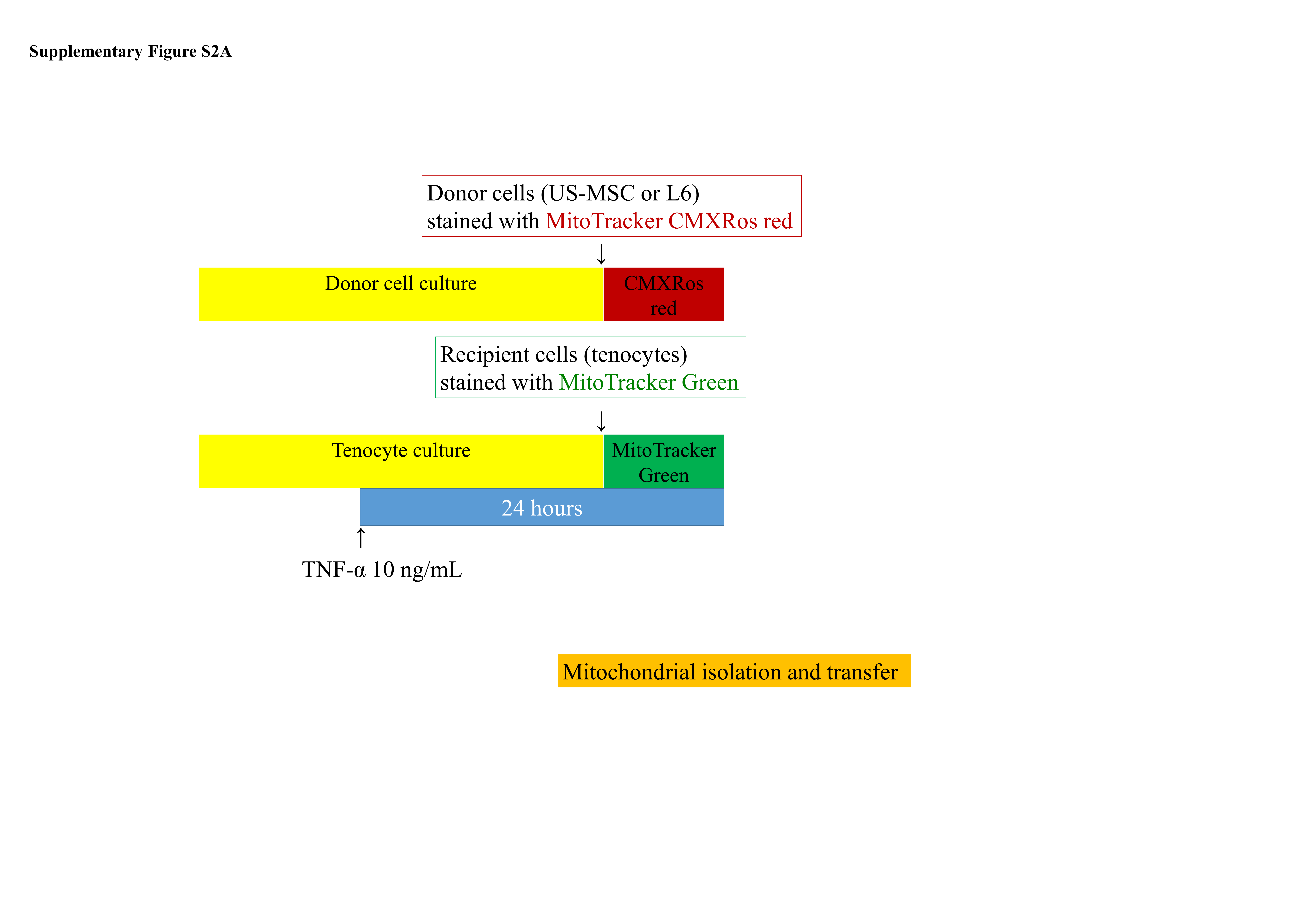

Supplement: Supplementary file 1 [file antioxidants-10-00696-s001.zip › antioxidants-1177808-supplementary/Supplementary Figures R2/Sup_Fig._S2(B)_R2.TIF]

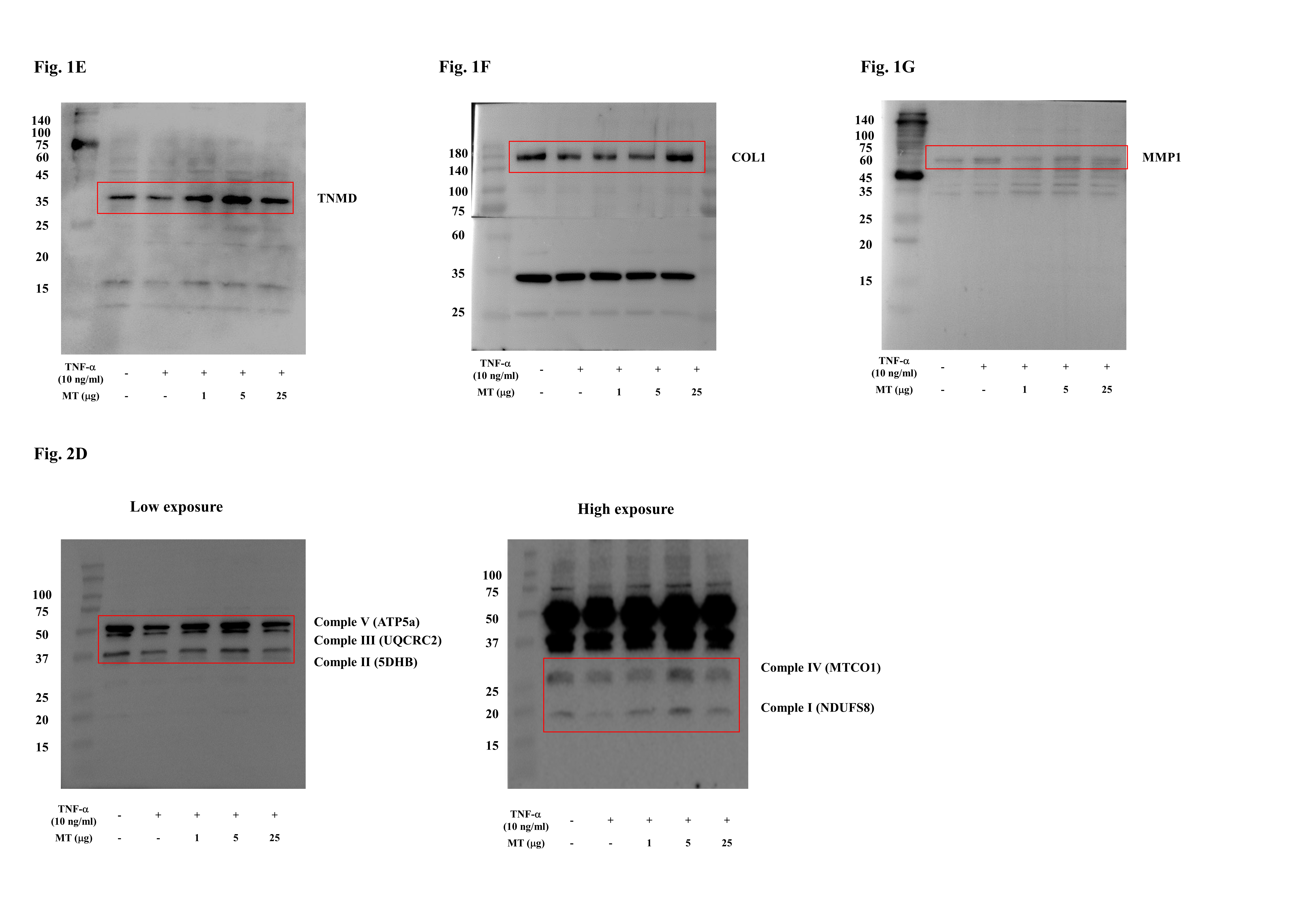

Supplement: Supplementary file 1 [file antioxidants-10-00696-s001.zip › antioxidants-1177808-supplementary/Supplementary Figures R2/Sup_Fig._S3(Fig.1_2)_R2.TIF]

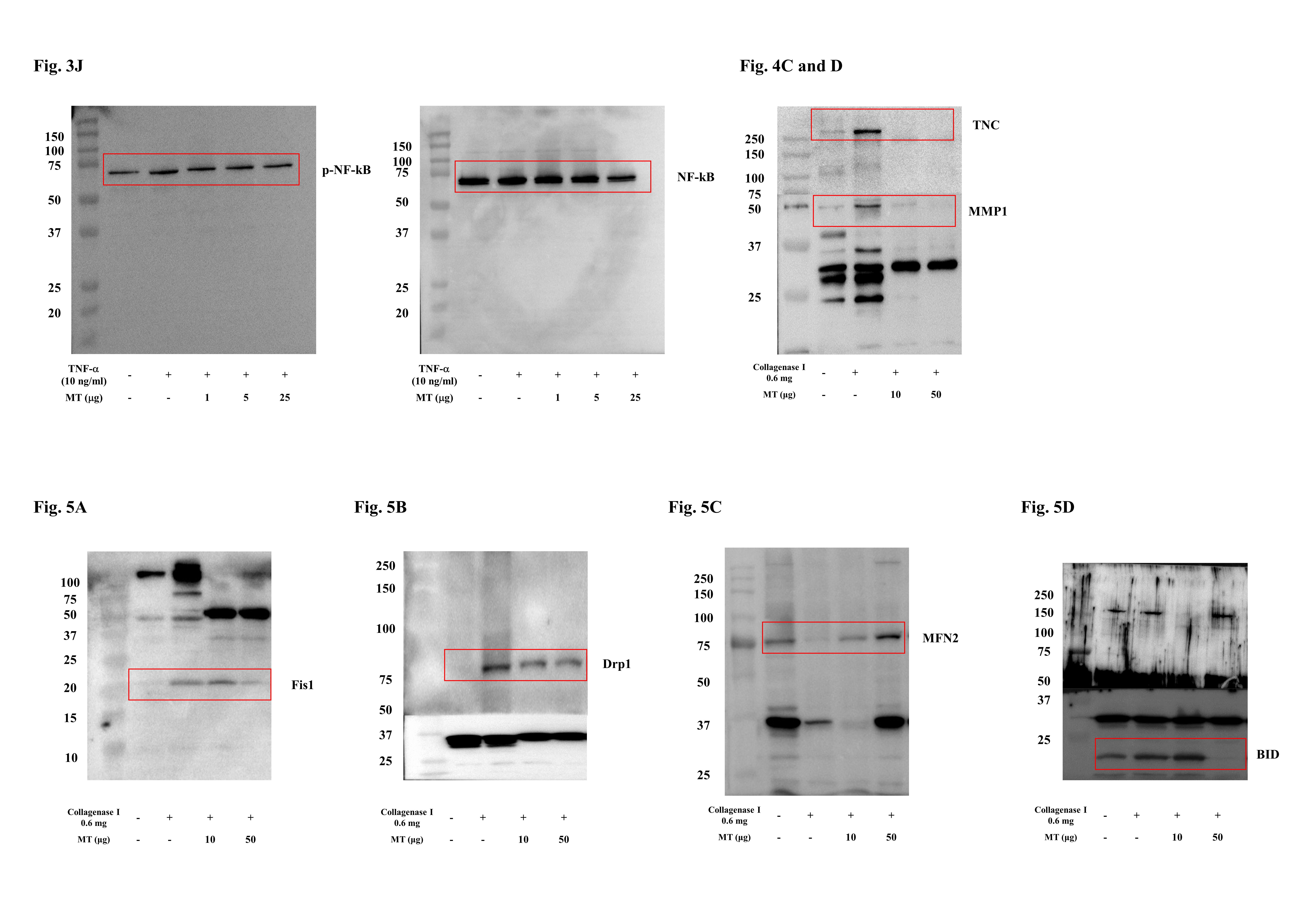

Supplement: Supplementary file 1 [file antioxidants-10-00696-s001.zip › antioxidants-1177808-supplementary/Supplementary Figures R2/Sup_Fig._S3(Fig.3J_4_5)_R2.TIF]

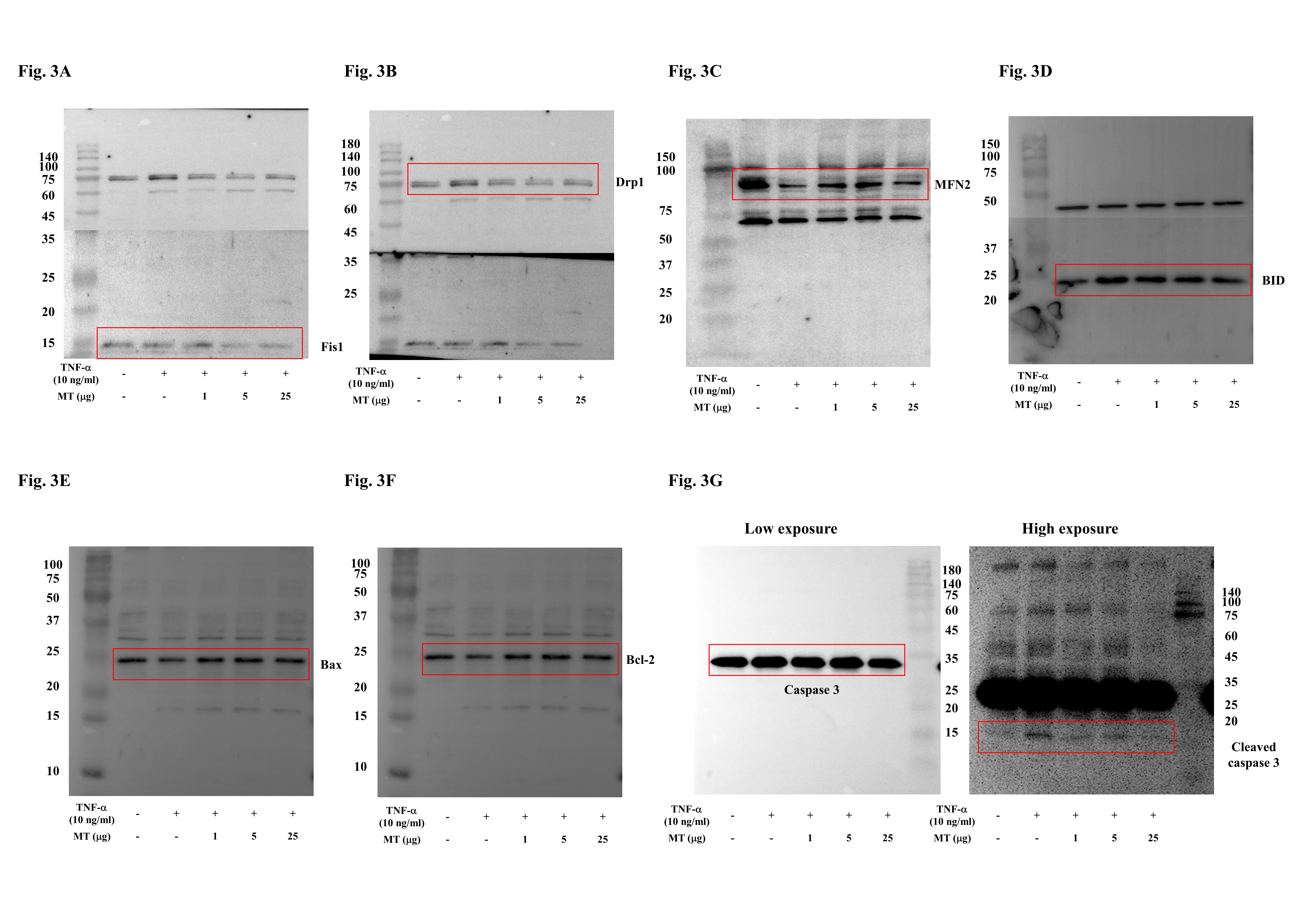

Supplement: Supplementary file 1 [file antioxidants-10-00696-s001.zip › antioxidants-1177808-supplementary/Supplementary Figures R2/Sup_Fig._S3(Fig.3_A-G)_R2.TIF]

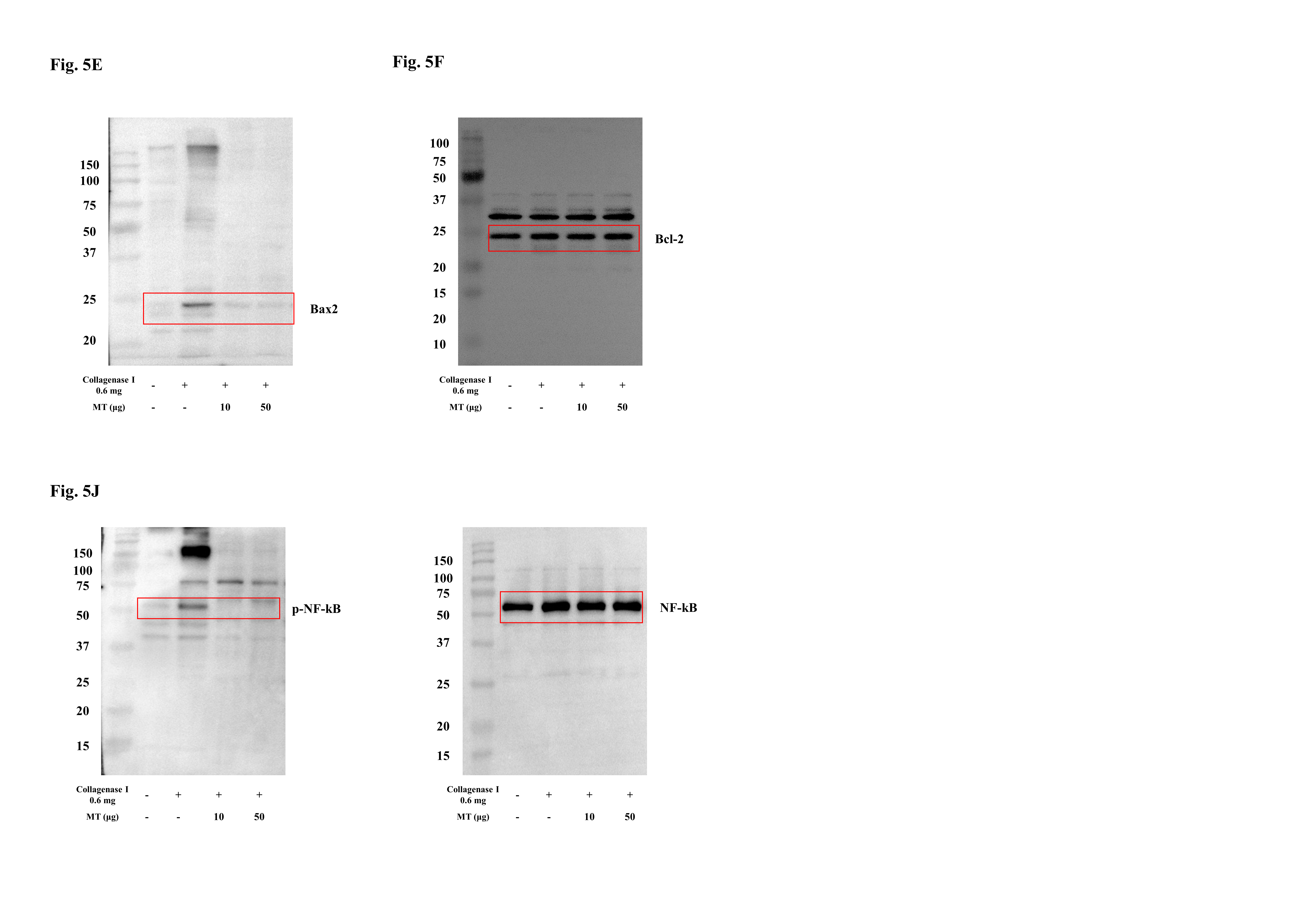

Supplement: Supplementary file 1 [file antioxidants-10-00696-s001.zip › antioxidants-1177808-supplementary/Supplementary Figures R2/Sup_Fig._S3(Fig.5_E-J)_R2.TIF]

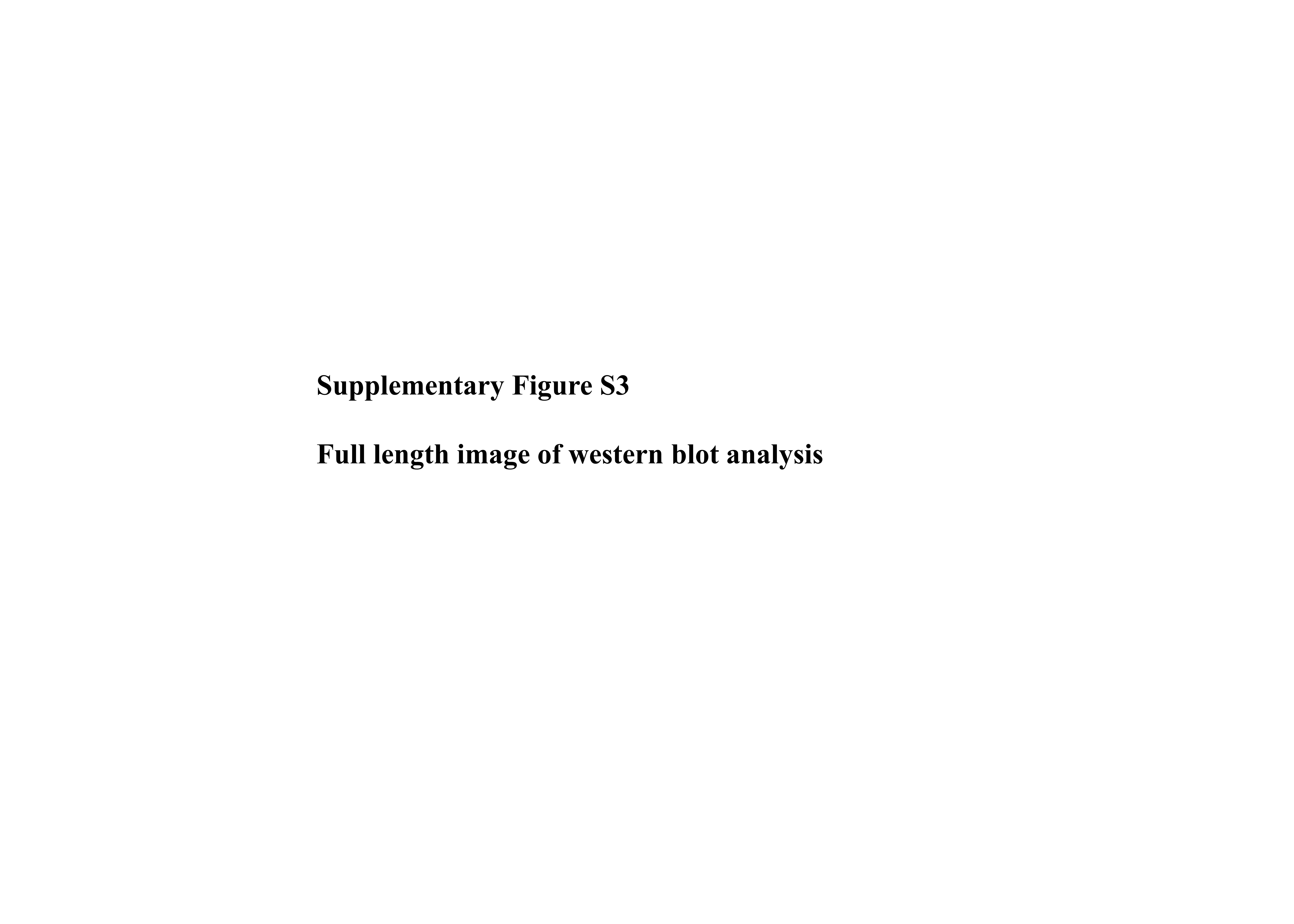

Supplement: Supplementary file 1 [file antioxidants-10-00696-s001.zip › antioxidants-1177808-supplementary/Supplementary Figures R2/Sup_Fig._S3_Title_R2.TIF]

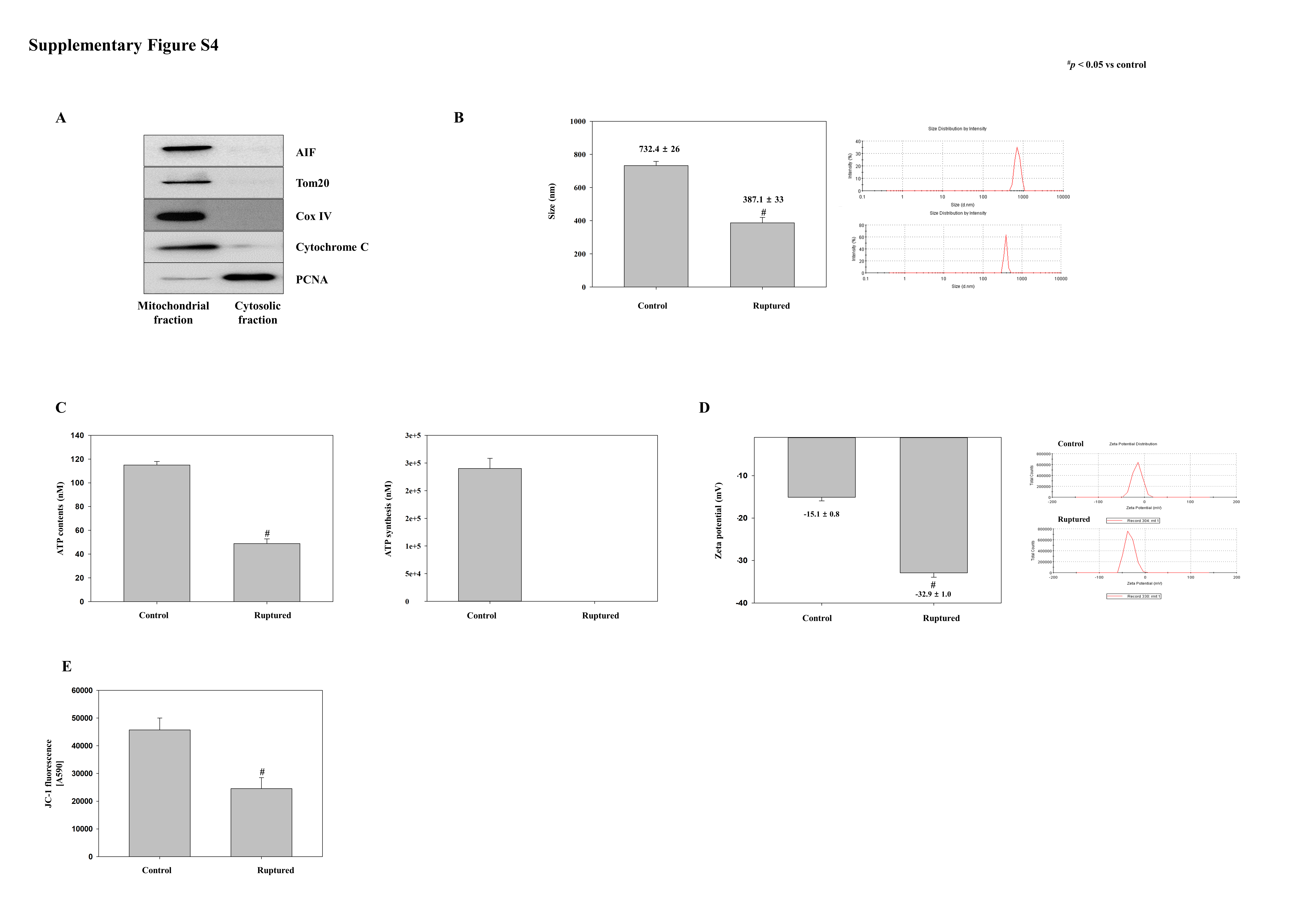

Supplement: Supplementary file 1 [file antioxidants-10-00696-s001.zip › antioxidants-1177808-supplementary/Supplementary Figures R2/Sup_Fig._S4_R2.TIF]

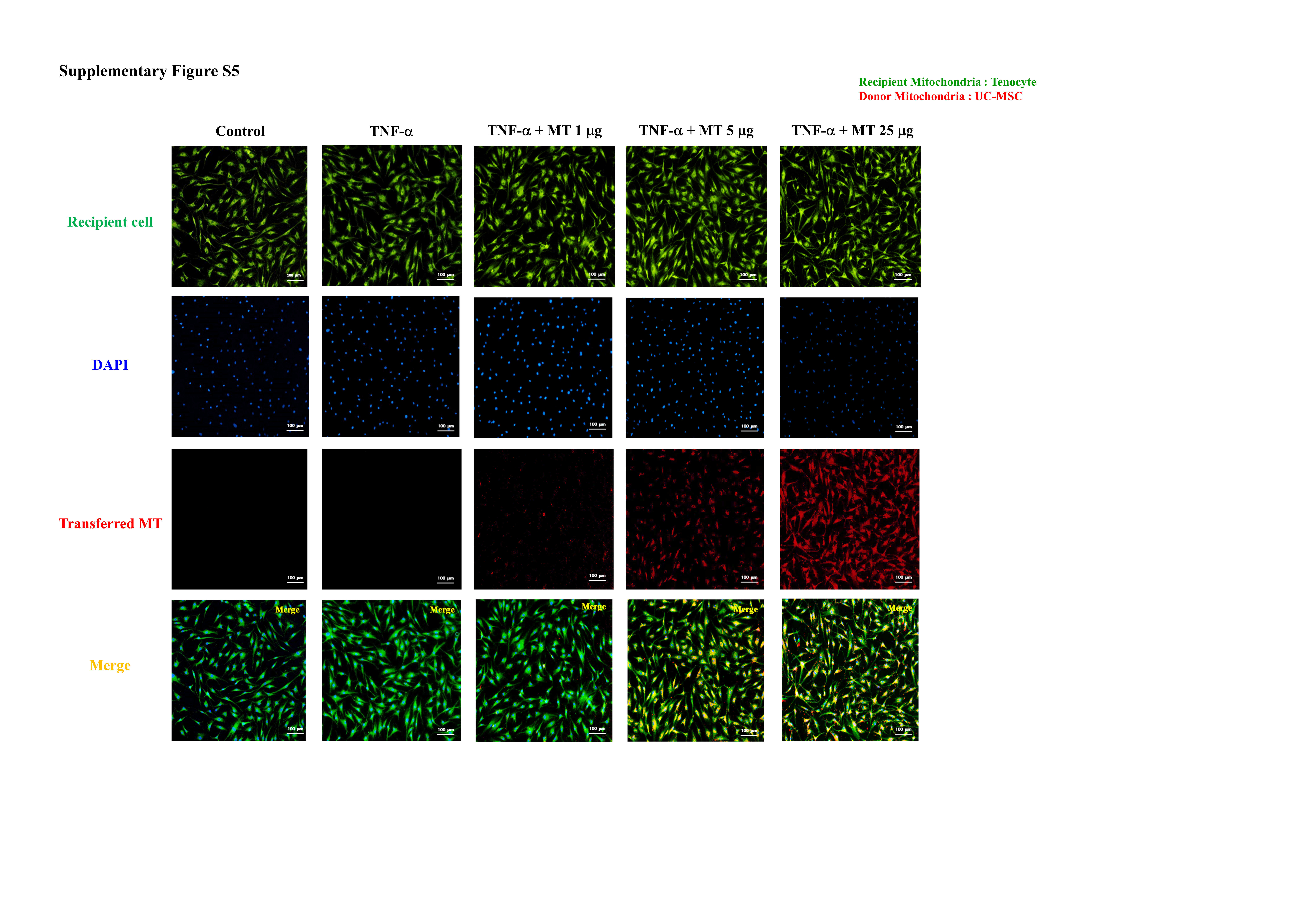

Supplement: Supplementary file 1 [file antioxidants-10-00696-s001.zip › antioxidants-1177808-supplementary/Supplementary Figures R2/Sup_Fig._S5_R2.TIF]

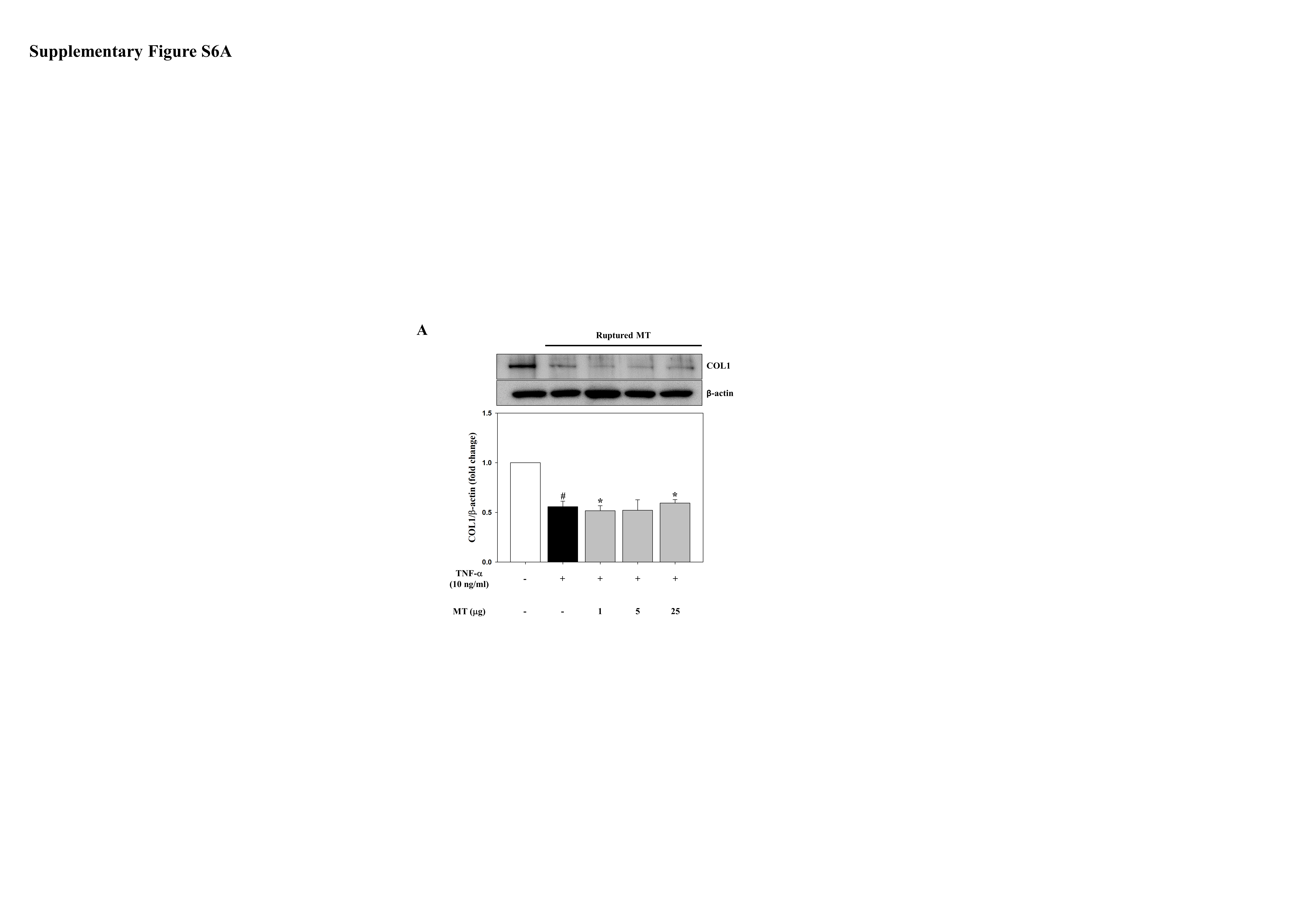

Supplement: Supplementary file 1 [file antioxidants-10-00696-s001.zip › antioxidants-1177808-supplementary/Supplementary Figures R2/Sup_Fig._S6(A)_R2.TIF]

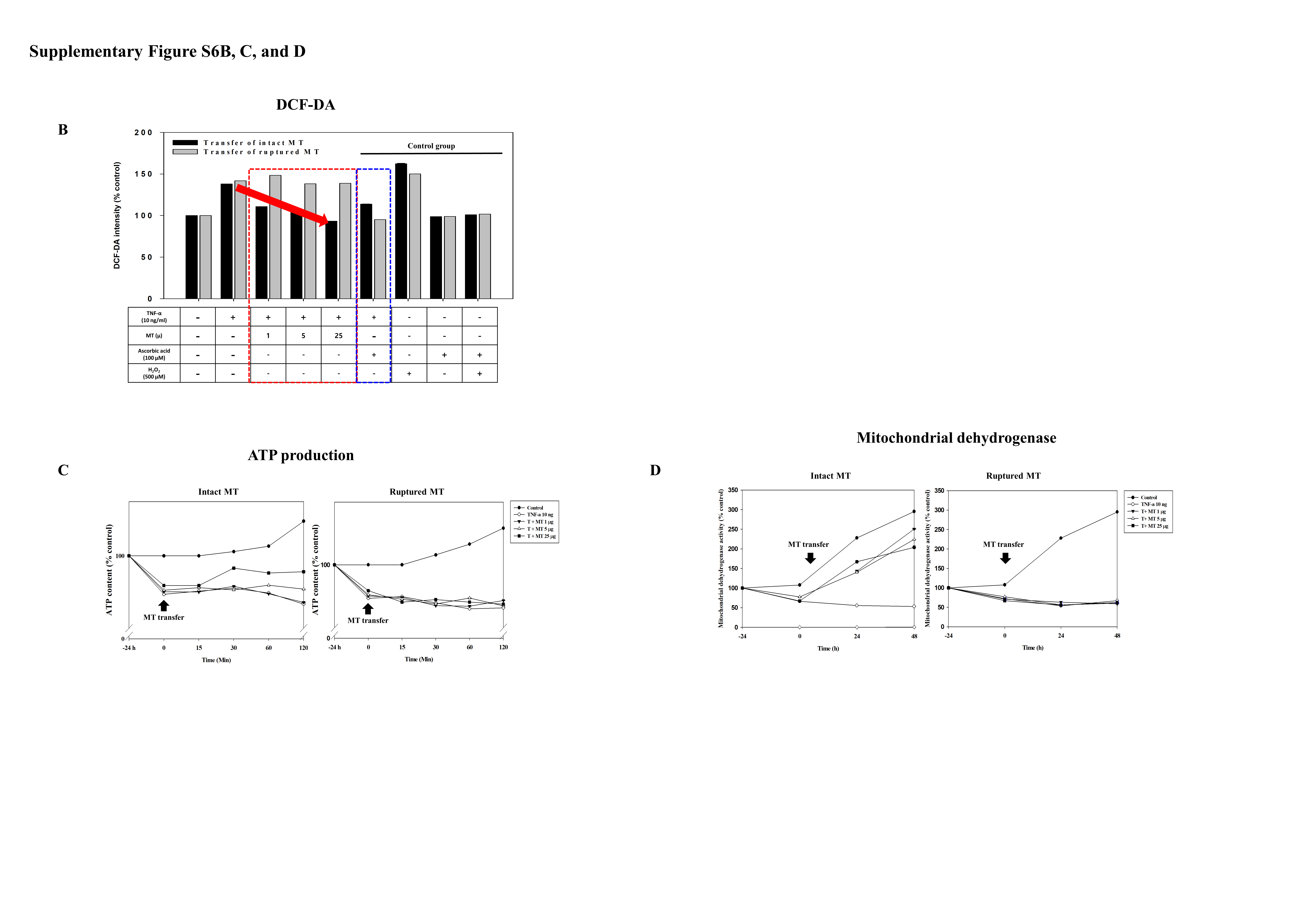

Supplement: Supplementary file 1 [file antioxidants-10-00696-s001.zip › antioxidants-1177808-supplementary/Supplementary Figures R2/Sup_Fig._S6(B-D)_R2.TIF]

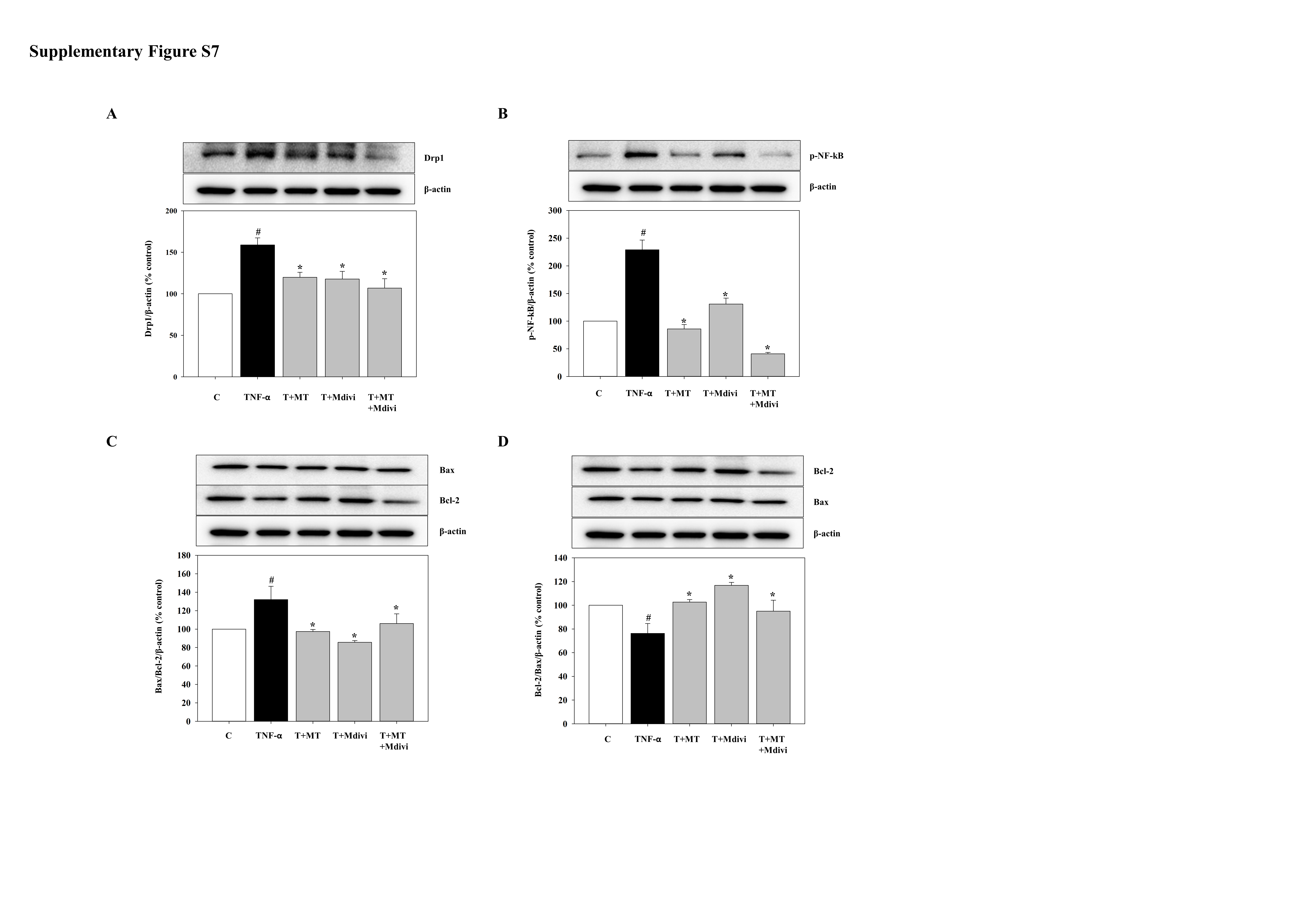

Supplement: Supplementary file 1 [file antioxidants-10-00696-s001.zip › antioxidants-1177808-supplementary/Supplementary Figures R2/Sup_Fig._S7_R2.TIF]

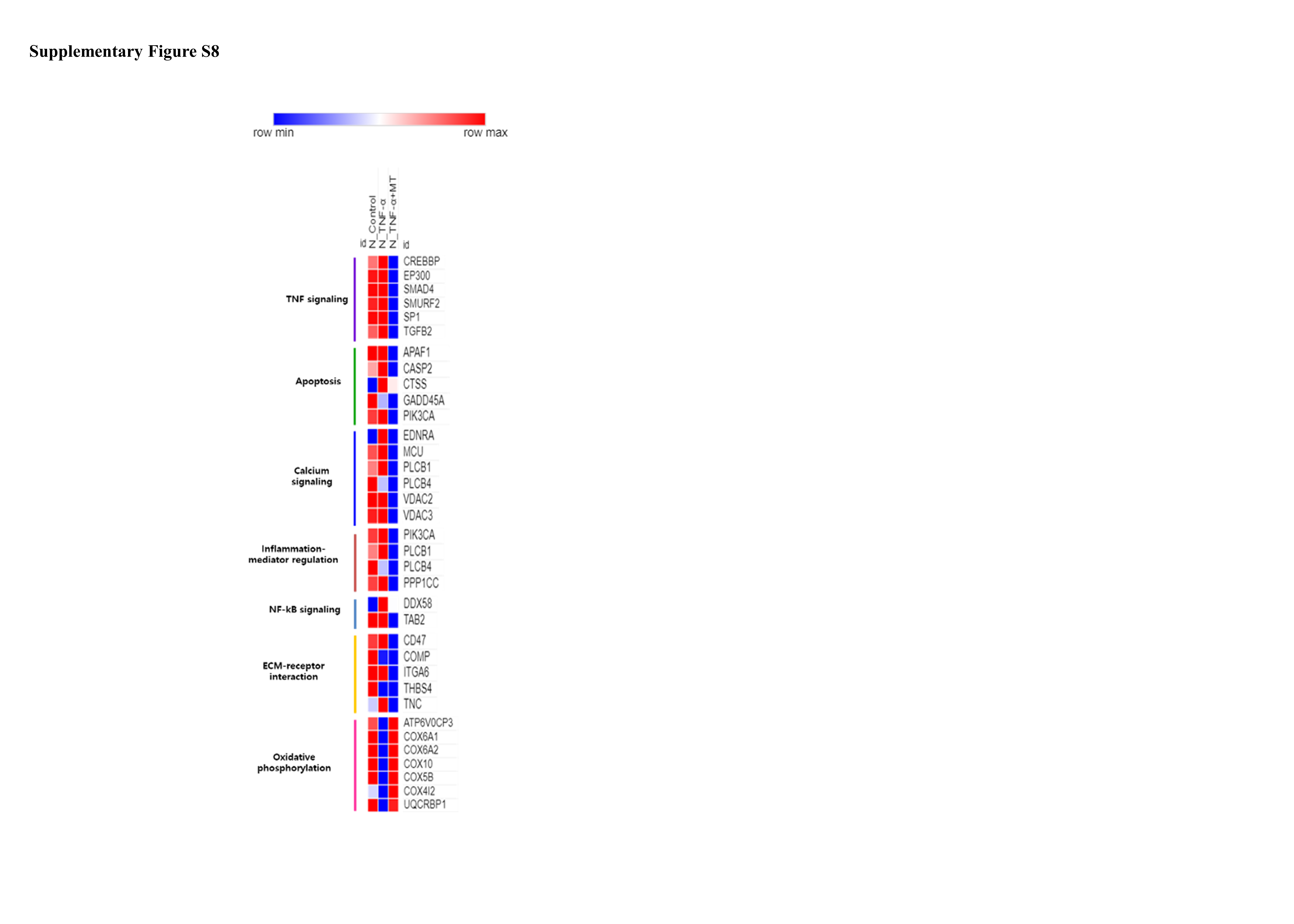

Supplement: Supplementary file 1 [file antioxidants-10-00696-s001.zip › antioxidants-1177808-supplementary/Supplementary Figures R2/Sup_Fig._S8_R2.TIF]

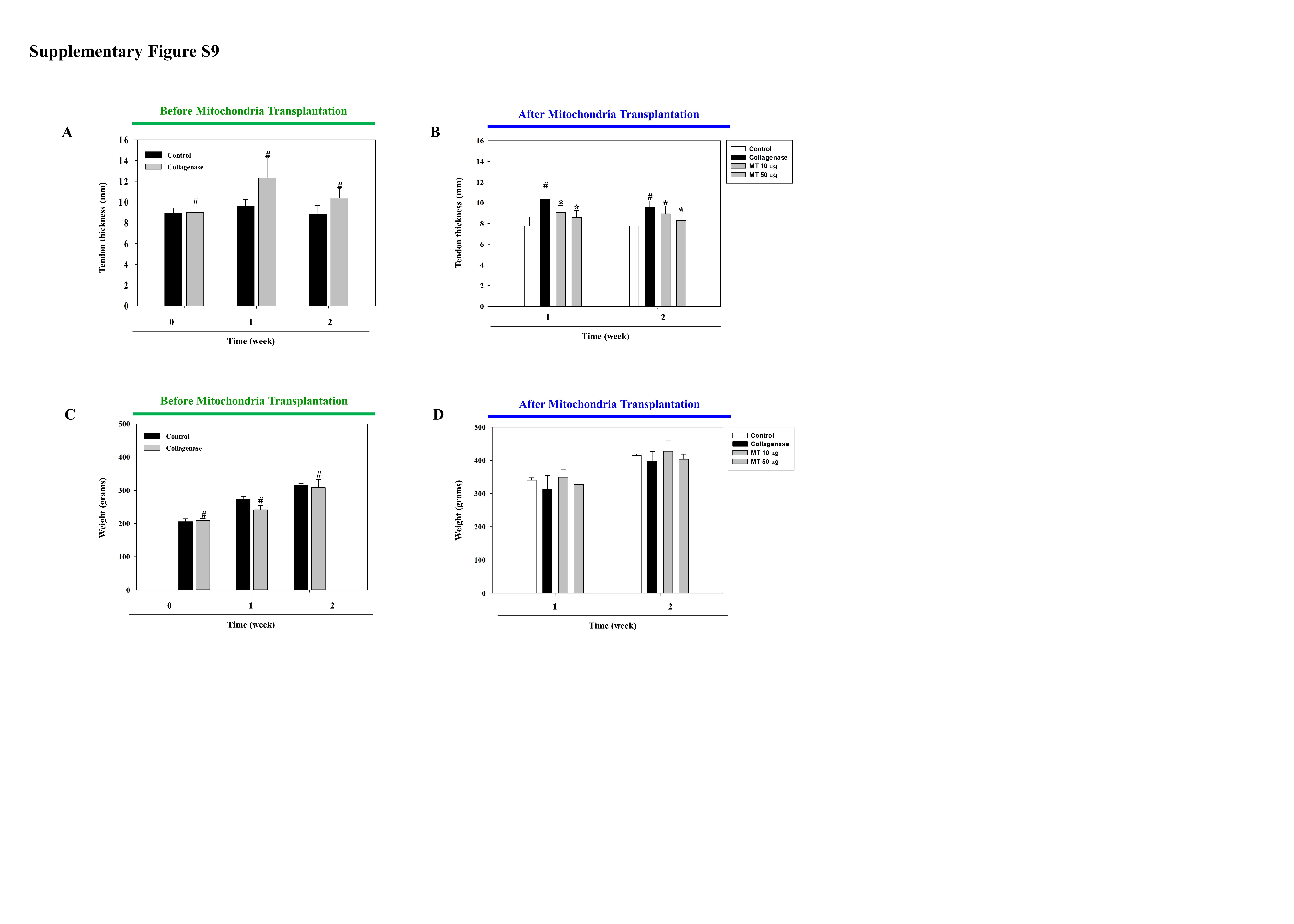

Supplement: Supplementary file 1 [file antioxidants-10-00696-s001.zip › antioxidants-1177808-supplementary/Supplementary Figures R2/Sup_Fig._S9_R2.TIF]

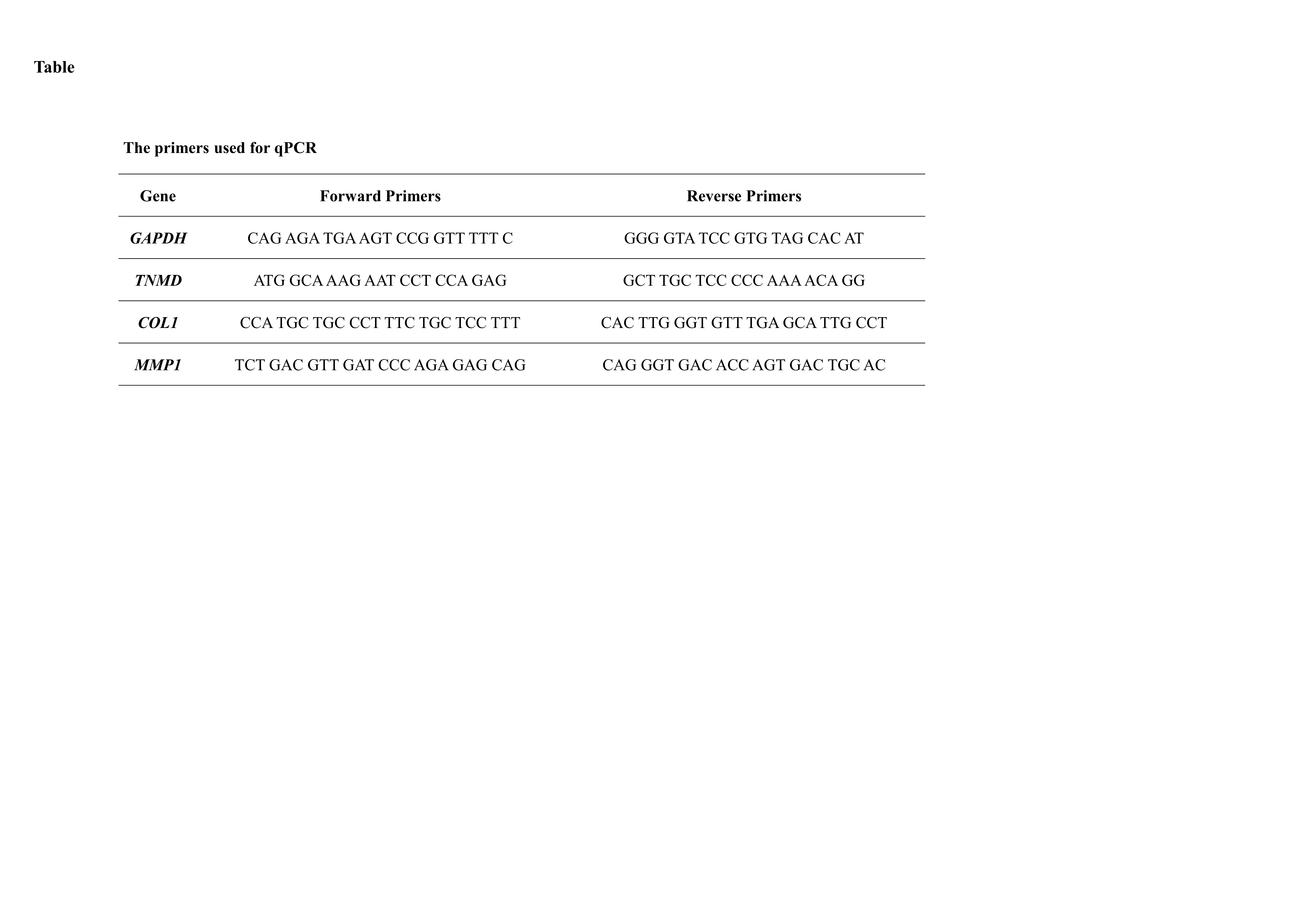

Supplement: Supplementary file 1 [file antioxidants-10-00696-s001.zip › antioxidants-1177808-supplementary/Supplementary Figures R2/Sup_Table.TIF]
